# A single parameter can predict surfactant impairment of superhydrophobic drag reduction

**DOI:** 10.1073/pnas.2211092120

**Published:** 2023-01-12

**Authors:** Fernando Temprano-Coleto, Scott M. Smith, François J. Peaudecerf, Julien R. Landel, Frédéric Gibou, Paolo Luzzatto-Fegiz

**Affiliations:** ^a^Department of Mechanical Engineering, University of California, Santa Barbara, CA 93106; ^b^Institute of Environmental Engineering, Department of Civil, Environmental and Geomatic Engineering, Eidgenössische Technische Hochschule (ETH) Zürich 8093, Zürich, Switzerland; ^c^Department of Mathematics, Alan Turing Building, University of Manchester, Manchester M13 9PL, United Kingdom

**Keywords:** superhydrophobic surface, drag reduction, surfactant, Marangoni stress, plastron

## Abstract

Recent experimental and computational investigations have shown that trace amounts of surfactants, unavoidable in practice, can critically impair the drag reduction of superhydrophobic surfaces (SHSs), by inducing Marangoni stresses at the air–liquid interface. However, predictive models for realistic SHS geometries do not yet exist, which has limited the understanding and mitigation of these adverse surfactant effects. To address this issue, we derive a model for laminar, three-dimensional flow over SHS gratings as a function of geometry and soluble surfactant properties, which together encompass 10 dimensionless groups. We establish that the grating length *g* is the key geometric parameter and predict that the ratio between actual and surfactant-free slip increases with *g*^2^. Guided by our model, we perform synergistic numerical simulations and microfluidic experiments, finding good agreement with the theory as we vary surfactant type and SHS geometry. Our model also enables the estimation, based on velocity measurements, of a priori unknown properties of surfactants inherently present in microfluidic systems. For SHSs, we show that surfactant effects can be predicted by a single parameter, representing the ratio between the grating length and the interface length scale beyond which the flow mobilizes the air–water interface. This mobilization length is more sensitive to the surfactant chemistry than to its concentration, such that even trace-level contaminants may significantly increase drag if they are highly surface active. These findings advance the fundamental understanding of realistic interfacial flows and provide practical strategies to maximize superhydrophobic drag reduction.

## Significance

Trace surfactants, unavoidable in applications, can impair the drag reduction achieved by superhydrophobic surfaces (SHS) as Marangoni stresses immobilize the air–water interface. It is not known how SHS impairment depends on surfactant type and concentration, flow velocity, and SHS geometry; as a result, mitigation strategies are still needed. We introduce a model of this phenomenon and perform simulations and experiments. We find that the interface can be mobilized if it is longer than a critical length scale, which is determined by the surfactant properties, essentially independently of flow velocity. SHS impairment is thereby predicted from a single parameter, namely the ratio of interface length and mobilization scale, providing fundamental insight and practical guidance to achieve superhydrophobic drag reduction.

Superhydrophobic surfaces (SHSs) have the potential to yield enormous technological benefits in fields ranging from microfluidics to maritime transportation, primarily due to their ability to reduce drag (1). Through a combination of hydrophobic chemistry and microscopic surface patterning, these substrates are able to retain a superficial layer of air, thereby producing an apparent slip when in contact with a liquid flow (2). Early theoretical work (3–5) modeled the air pockets trapped within these textures as flat boundaries with no shear, predicting large drag reductions in the laminar regime. Although early experiments found promising levels of drag reduction (6–9), subsequent studies measured a reduced or even nonexistent slip (10–12), pointing at the interfacial stresses induced by surface-active contaminants as one possible cause of this discrepancy. Recently, independent experimental studies have reported time-dependent and spatially complex interfacial dynamics that unequivocally demonstrate the importance of surfactant-induced stresses on SHSs (13, 14). Theoretical and computational works have confirmed the extent to which trace amounts of these surface-active contaminants can reduce slip (15–18). This slip reduction is also consistent with broader findings for small-scale multiphase flows, where environmental levels of surfactants, often extremely difficult to avoid or control, play a central role (19); prominent examples are given by small bubbles rising in water (e.g., refs. (20–23) and references therein) or bubbles probed by atomic force microscopy (24, 25). These flows have been understood through models that include surfactants, sometimes at trace levels that are undetectable by traditional surface tension measurements.

**Fig. 1.**
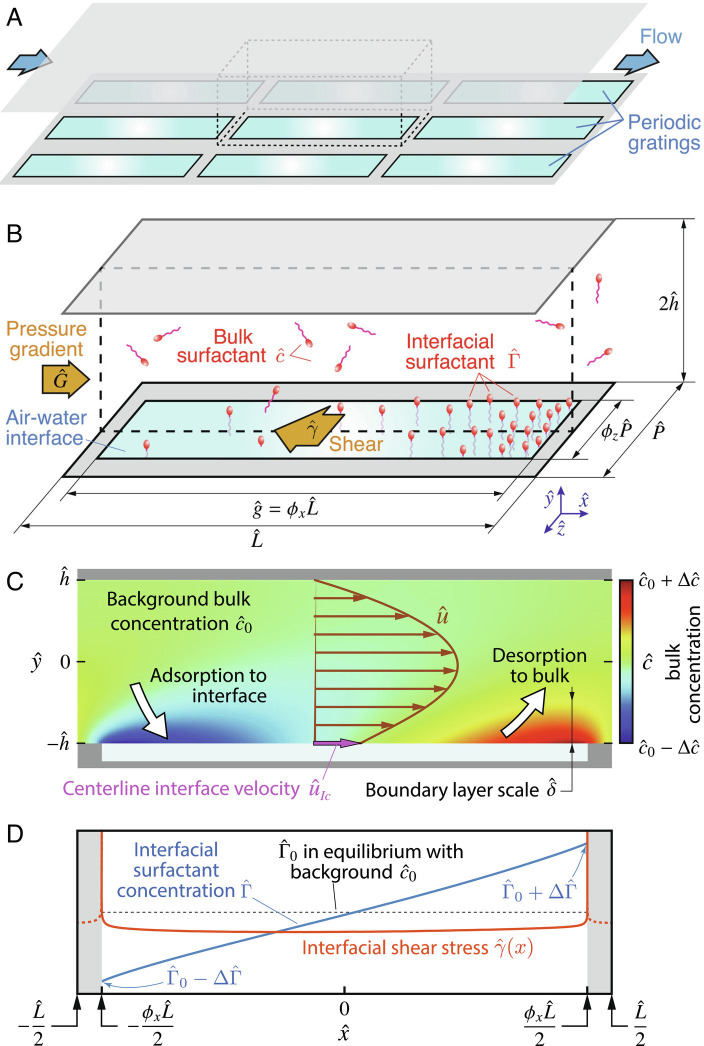
(*A*) Diagram showing the plane channel flow arrangement studied, with an array of slender periodic streamwise gratings on the bottom wall. (*B*) Unit cell of the SHS (also shown with dashed lines in *panel* *A*), periodic in $\hat{x}$ and $\hat{z}$, illustrating the downstream accumulation of surfactant. (*C*) Streamwise cross-section at mid-grating ($\hat{z}=0$, also shown with dashed lines in *panel* *B*), showing a field of bulk surfactant from a representative simulation, with adsorption/desorption regions at the upstream/downstream ends, respectively. (*D*) Interface concentration (blue) and shear stress (red), for the same $\hat{z}=0$ cross-section. Throughout the article, hats denote dimensional quantities.

For SHS textures, the concentration gradients that induce Marangoni stresses appear in the streamwise direction, owing to stagnation points at the downstream ends of the interfaces, where advected surfactants accumulate (Fig. 1 *A* and *B*). Modeling this physical mechanism for realistic SHS geometries is challenging. In addition to the four coupled partial differential equations governing the physics and the ten associated dimensionless numbers (detailed below), there is a major difficulty stemming from the alternating slip/no-slip boundary conditions at the edges where the fluid interface meets the solid substrate. The resulting spatially complex flows constitute a challenge for analytical progress. For this reason, models with surfactants considered only two-dimensional (2D) flows over transverse SHS gratings as this is the simplest geometry that captures detrimental surfactant effects (15, 17). A more realistic configuration is that of streamwise gratings, which are widely used (6, 11, 13, 26), owing to their potential for very high drag reduction in surfactant-free conditions. Gratings have been modeled as infinitely long in surfactant-free theories (3, 5, 27–29); however, modeling surfactant effects requires considering finite streamwise gratings with stagnation points, leading to a three-dimensional (3D) flow. Theories of realistic gratings inclusive of surfactant are still needed.

Here, we introduce a theory for 3D flow over streamwise SHS gratings with surfactants, by coupling a hydrodynamic solution (for slender, finite gratings with arbitrary shear at the interface) with a scaling analysis of surfactant dynamics (for soluble, dilute surfactants). We use our model to design experiments and simulations where the slip velocity varies across three orders of magnitude, relative to surfactant-free conditions, and thereby achieve a direct comparison between theory and experiments for realistic SHSs. Our theory can also use velocity measurements to estimate physicochemical parameters of unknown, trace-level surfactants, which are inevitable both in natural and artificial settings. Although the general problem comprises ten dimensionless groups, we show that impairment by trace surfactant is approximately controlled by a single parameter, which depends on surfactant type and concentration, and is independent of flow velocity. Since surface-active molecules are naturally released by polymers widely used in microfabrication (30–33), we expect these results to be valuable over a broad range of fundamental and applied microfluidic research.

## SHS Model for 3D Flow with Surfactants

We consider steady, laminar flow driven by a mean pressure gradient $\hat{G}$ across a channel of half-height $\hat{h}$, where hats denote dimensional quantities. The bottom of the channel is lined with a periodic pattern of slender, rectangular gratings (Fig. 1*A*). Each gas–liquid interface (the “plastron”) is assumed flat. Due to the periodicity of the array in the streamwise and spanwise directions, we focus on a unit cell consisting of one grating and its surrounding ridges, as depicted in Fig. 1*A*. The streamwise, wall-normal, and spanwise directions are $\hat{x}$, $\hat{y}$, and $\hat{z}$, respectively, with the coordinate origin at the center of the unit cell (Fig. 1*B*).

We leverage the disparity of scales between the length $\hat{L}$ and the half-height $\hat{h}$ (Fig. 1*B*) and define a small parameter $\varepsilon =\hat{h}/\hat{L}\ll 1$, which is dimensionless and thus written without hats. Differently from the classic Hele-Shaw flow approximation (28), here we do not assume that the spanwise length scale (the pitch $\hat{P}$) is much larger than $\hat{h}$, since, in microfluidic applications, $\hat{h}$ and $\hat{P}$ are of the order of tens of micrometers, whereas $\hat{L}$ ranges in the millimeter or centimeter scale (6, 7, 11, 26). Consequently, we define the nondimensional coordinates $x=\hat{x}/\hat{L}$, $y=\hat{y}/\hat{h}=\hat{y}/\left(\varepsilon \hat{L}\right)$, and $z=\hat{z}/\left(\varepsilon \hat{L}\right)$. Incompressibility implies that the flow is approximately unidirectional, with the dominant streamwise velocity scaling as $\hat{u}∼\hat{U}$, whereas the wall-normal and spanwise components scale as $\hat{v}∼\varepsilon \hat{U}$ and $\hat{w}∼\varepsilon \hat{U}$. The velocity scale is $\hat{U}={\hat{h}}^{2}\hat{G}/\hat{\mu }$, with $\hat{\mu }$ the dynamic viscosity. At leading order in *ε*, the Stokes equations for the flow are ∂_*yy*_*u* + ∂_*zz*_*u* = ∂_*x*_*p* and ∂_*y*_*p* = ∂_*z*_*p* = 0 (*SI Appendix*, *Flow Field Derivation*), where $u\left(x,y,z\right)=\hat{u}/\hat{U}$ and $p\left(x\right)=\hat{p}/\left(\hat{G}\hat{L}\right)$ are the dimensionless streamwise velocity and pressure. The unidirectional nature of this leading-order flow is a good approximation far from the downstream and upstream edges of the plastron, i.e., where |*x* ± *ϕ*_*x*_/2|≫*ε*, with *ϕ*_*x*_ the streamwise gas fraction (Fig. 1*B*). Therefore, the asymptotic expansion in *ε* is singular as is common for thin-gap approximations (34). Since we consider slender gratings with *ε* ≪ 1, the regions of validity represent most of the domain, and useful approximations of both local and integrated quantities can be obtained.

No-slip boundary conditions *u* = 0 are imposed at solid walls and ridges. The interface imposes a Marangoni stress $\hat{\gamma }$ determined by the local gradient of interfacial (adsorbed) surfactant. This stress is independent of transverse direction *z* at leading order in *ε* (*SI Appendix*, *Flow Field Derivation*), and thus, the plastron boundary condition is ∂_*y*_*u*|_*I*_ = *γ*(*x*), where $\gamma (x)=\hat{\gamma }/\left(\hat{\mu }\hat{U}/\varepsilon \hat{L}\right)$ and the subscript *I* denotes conditions at the interface.

Note that, for finite gratings, the pressure gradient is not constant in *x*, and *p*(*x*) must be determined from two integral constraints. First, the volume flow rate $Q=\int_{-P/2}^{P/2}\int_{-1}^{1}u\left(x,y,z\right)\;dy\;dz$ (where $P=\hat{P}/\hat{h}$ is the normalized pitch; Fig. 1*B*) must be independent of *x* to satisfy mass conservation. Second, the pressure drop across the whole unit cell must match the imposed mean pressure gradient, such that $\int_{-1/2}^{1/2}\partial_{x}p\left(x\right)\;dx=-1$. These two conditions lead to an expression for the flow field (as detailed in *SI Appendix*, *Flow Field Derivation*),

[1] $$\begin{array}{c} u\left(x,y,z\right)=\{\begin{array}{c} \frac{[1+q_{d}^{\infty }\left(1-\phi_{x}\right)\left\langle \gamma \rangle \right]u_{P}\left(y\right)+[1-\langle \gamma \rangle ]u_{d}^{\infty }\left(y,z\right)}{1+q_{d}^{\infty }\left(1-\phi_{x}\right)}\\ -\frac{\gamma (x)-\langle \gamma \rangle }{1+q_{d}^{\infty }}[q_{d}^{\infty }u_{P}\left(y\right)-u_{d}^{\infty }\left(y,z\right)],\;\;\text{if}\left|x\right|<\frac{\phi_{x}}{2}\\ {}[\frac{1+q_{d}^{\infty }\left(1-\phi_{x}\left\langle \gamma \right\rangle \right)}{1+q_{d}^{\infty }\left(1-\phi_{x}\right)}]u_{P}\left(y\right),\;\;\text{if}\frac{\phi_{x}}{2}<\left|x\right|\leq \frac{1}{2}, \end{array} \end{array}$$

where *u*_*P*_(*y*)=(1 − *y*^2^)/2 is the Poiseuille profile and *u*_*d*_^∞^(*y*, *z*) is the deviation from *u*_*P*_(*y*) in the infinite-grating case, where the interface has no stagnation points and thus surfactant effects are absent. In other words, if *ϕ*_*x*_ = 1 then *u*(*y*, *z*)=*u*_*P*_(*y*)+*u*_*d*_^∞^(*y*, *z*) and *γ*(*x*) = 0, where *u*_*d*_^∞^(*y*, *z*) is known from previous studies (3, 35). In [], *q*_*d*_^∞^ = 3*Q*_*d*_^∞^/(2*P*), where $Q_{d}^{\infty }=\int_{-P/2}^{P/2}\int_{-1}^{1}u_{d}^{\infty }\left(y,z\right)\;dy\;dz$, and $\left\langle \gamma \right\rangle =\frac{1}{\phi_{x}}\int_{-\phi_{x}/2}^{\phi_{x}/2}\gamma (x)\;dx$ is the average Marangoni shear across the plastron, which varies between 0, for a clean interface, and 1 for a fully immobilized interface. Eq. **1** provides the velocity field as a linear combination of two known simpler solutions (*u*_*P*_ and *u*_*d*_^∞^), relying on parameters which are either prescribed (*γ*(*x*)) or known from the infinite-grating problem (*q*_*d*_^∞^).

The flow field is linked to the surfactant dynamics via *γ*, which is found from the equations for soluble surfactant (*SI Appendix*, *Governing Equations*)

[2a] $$\begin{array}{cc} & u\frac{\partial^{c}}{\partial x}+v\frac{\partial^{c}}{\partial y}+w\frac{\partial^{c}}{\partial z}=\frac{1}{\varepsilon \mathrm{Pe}}(\varepsilon^{2}\frac{\partial^{2}c}{\partial x^{2}}+\frac{\partial^{2}c}{\partial y^{2}}+\frac{\partial^{2}c}{\partial z^{2}}), \end{array}$$

[2b] $$\begin{array}{cc} & \frac{\partial^{(}u\Gamma )}{\partial x}+\frac{\partial^{(}w\Gamma )}{\partial z}=\frac{1}{\varepsilon Pe_{I}}(\varepsilon^{2}\frac{\partial^{2}\Gamma }{\partial x^{2}}+\frac{\partial^{2}\Gamma }{\partial z^{2}})+\frac{\mathrm{Bi}}{\varepsilon }(c_{I}-\Gamma ), \end{array}$$

[2c] $$\begin{array}{cc} & \frac{\partial^{c}}{\partial y}{\text{|}}_{I}=Da\left(c_{I}-\text{Γ}\right)\;\text{at the interface}, \end{array}$$

[2d] $$\begin{array}{cc} & \frac{\partial^{u}}{\partial y}{\text{|}}_{I}=\gamma (x)=\varepsilon kMa\frac{\partial^{\text{Γ}}}{\partial x}\;\;\text{at the interface}. \end{array}$$

Eqs. **2a** and **2b** describe the transport of the nondimensional bulk and interfacial surfactant concentrations $c=\hat{c}/{\hat{c}}_{0}$ and $\Gamma =\hat{\Gamma }/{\hat{\Gamma }}_{0}$, respectively, where ${\hat{c}}_{0}$ is the background bulk concentration and ${\hat{\Gamma }}_{0}$ is the equilibrium interfacial concentration (Fig. 1 *C* and *D*). The adsorption and desorption kinetics are modeled through **2c** and the last term in 2b, whereas the Marangoni boundary condition **2d** relates the shear stress to the gradient of surfactant concentration at the interface. Six dimensionless groups control the surfactant dynamics in Eq. **2**. The bulk and interface Péclet numbers are $Pe=\hat{h}\hat{U}/\hat{D}$ and $Pe_{I}=\hat{h}\hat{U}/{\hat{D}}_{I}$, where $\hat{D}$ and ${\hat{D}}_{I}$ are the bulk and interface diffusivities. The Marangoni number $Ma=n_{s}\hat{R}\hat{T}{\hat{\Gamma }}_{m}/\left(\hat{\mu }\hat{U}\right)$ depends on the maximum interfacial packing concentration ${\hat{\Gamma }}_{m}$, the ideal gas constant $\hat{R}$, the temperature $\hat{T}$, and a parameter *n*_*s*_ quantifying the effects of salinity. The Biot $Bi=\hat{h}{\hat{\kappa }}_{d}/\hat{U}$ and Damköhler $Da=\hat{h}{\hat{\kappa }}_{a}{\hat{\Gamma }}_{m}/\hat{D}$ numbers parameterize the effect of kinetics, with ${\hat{\kappa }}_{a}$ and ${\hat{\kappa }}_{d}$ being the adsorption and desorption rate constants. The normalized background concentration is $k={\hat{\kappa }}_{a}{\hat{c}}_{0}/{\hat{\kappa }}_{d}$ and can be related to the interfacial concentration at kinetic equilibrium through $k={\hat{\Gamma }}_{0}/{\hat{\Gamma }}_{m}$. These six dimensionless groups, in addition to four geometrical parameters *ϕ*_*x*_, *ϕ*_*z*_, *P*, and $g=\hat{g}/\hat{h}=\phi_{x}/\varepsilon$, fully describe the flow and surfactant transport problem.

A scaling analysis of Eq. **2**, similar to the one performed in ref. (15) for transverse gratings, leads to an expression for ⟨*γ*⟩. The derivation, which can be found in *SI Appendix*, *Scaling Theory for Surfactant Transport*, is based on the assumption of low normalized concentration (*k* ≪ 1), which justifies the choice of Henry kinetics (19) in Eq. **2**, and is the case in applications unless substantial amounts of surfactant are deliberately added (12–14). The stress is approximated as spatially uniform, i.e., *γ*(*x*) ≈ ⟨*γ*⟩, as has been found to be the case in small-scale applications (15) such that the term on the second line of Eq. **1** is negligible.

To quantify slip and enable comparison with experiments, we use the centerline slip velocity *u*_*Ic*_, defined as the velocity along the centerline of the interface, *u*_*Ic*_ = *u*(*x*, *y* = −1, *z* = 0), as illustrated in Fig. 1*C*. We use *u*_*Ic*_ because it can be measured with greater ease and accuracy than the local slip length *λ* = *u*_*I*_/∂_*y*_*u*|_*I*_, which requires estimation of velocity gradients (12). From the combination of the flow field from Eq. **1** and the expression for ⟨*γ*⟩ obtained from the scaling of Eq. **2**, we obtain

[3] $$\begin{array}{cc} \frac{u_{\mathrm{Ic}}}{u_{\mathrm{Ic}}^{\mathrm{clean}}} & =1-\frac{a_{1}\;k\;\mathrm{Ma}\;u_{\mathrm{Ic}}^{\mathrm{clean}}}{a_{1}\;k\;\mathrm{Ma}\;u_{\mathrm{Ic}}^{\mathrm{clean}}+a_{2}\frac{Bi\;g^{2}}{1+\delta \mathrm{Da}}+\frac{1}{Pe_{I}}}. \end{array}$$

Our model also yields an expression for the effective slip length *λ*_*e*_, defined through the wall-averaged Navier slip boundary condition that would result in the same flow rate as alternating no-slip/slip boundary conditions on the bottom wall (1, 5, 15, 35),

[4] $$\begin{array}{cc} \frac{\lambda_{e}}{\lambda_{e}^{\;}\text{clean}} & =1-\frac{a_{1}\;k\;\mathrm{Ma}\;u_{\mathrm{Ic}}^{\mathrm{clean}}(1+\frac{\lambda_{e}^{\mathrm{clean}}}{2})}{a_{1}\;k\;\mathrm{Ma}\;u_{\mathrm{Ic}}^{\mathrm{clean}}(1+\frac{\lambda_{e}^{\mathrm{clean}}}{2})+a_{2}\frac{Bi\;g^{2}}{1+\delta \mathrm{Da}}+\frac{1}{Pe_{I}}}. \end{array}$$

In Eqs. **3** and **4**, $\delta =\hat{\delta }/\hat{h}$ is the concentration boundary layer thickness (Fig. 1*C*), modeled as *δ*(*g*, *Pe*)=*a*_3_(1 + *a*_4_ *Pe*/*g*)^−1/3^ following a canonical Lévêque scaling (34). Here, *u*_*Ic*_^clean^ is the centerline slip velocity for *clean* finite-length gratings, found setting *γ*(*x*) = ⟨*γ*⟩ = 0 in Eq. **1**. This leads to *u*_*Ic*_^clean^ = *u*_*Ic*_^∞^/[1 + *q*_*d*_^∞^(1 − *ϕ*_*x*_)], where *u*_*Ic*_^∞^ is the centerline slip velocity for infinite gratings, known from previous studies (3, 35). The effective slip length for clean gratings is *λ*_*e*_^clean^ = 2*ϕ*_*x*_*q*_*d*_^∞^/[3 + *q*_*d*_^∞^(3 − 4*ϕ*_*x*_)]. Although exact solutions of *u*_*Ic*_^∞^ and *q*_*d*_^∞^ require numerical calculations, useful approximations are *u*_*Ic*_^∞^ ≈ {[(*P*/*π*)cosh^−1^(sec(*πϕ*_*z*_/2))]^*n*_*u*_^ + 2^*n*_*u*_^}^−1/*n*_*u*_^ and *q*_*d*_^∞^ ≈ {[(3*P*/2*π*)ln(sec(*πϕ*_*z*_/2))]^*n*_*q*_^ + (3*ϕ*_*z*_)^*n*_*q*_^}^−1/*n*_*q*_^, with *n*_*u*_ = −1.46, *n*_*q*_ = −1.21 (these become exact as *P* → 0 or *P* → ∞, *SI Appendix*, *Flow Field Derivation*).

Eqs. **3** and **4** link the loss of performance to the surfactant-induced stresses; these are enhanced by increasing the product of normalized concentration and Marangoni number *k* *Ma*, which expresses the ratio of Marangoni effects and viscous shear, for a unit of surfactant gradient. Marangoni stresses can be reduced by decreasing the surfactant gradient, achieved by either i) increasing surfactant flux between the interface and the bulk (relative to advection), as captured by *Bi* *g*^2^/(1 + *δ* *Da*), or ii) increasing interfacial diffusivity (again, compared to advection), expressed by 1/*Pe*_*I*_.

**Fig. 2.**
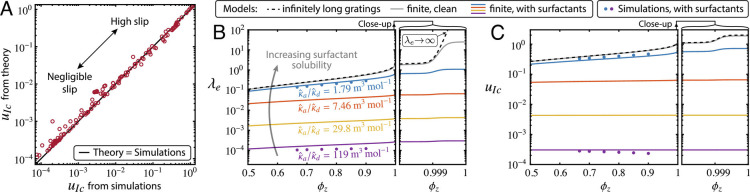
(*A*) Comparison of the centerline slip velocity from 155 numerical simulations with our model prediction Eq. **3**. (*B*) Effective slip length and (*C*) Centerline slip velocity as a function of the spanwise gas fraction *ϕ*_*z*_, for a fixed surfactant concentration ${\hat{c}}_{0}$ and varying surfactant solubility (${\hat{\kappa }}_{a}/{\hat{\kappa }}_{d}$). Unless noted, the parameters are as in *SI Appendix*, Table SI.

The scaling coefficients *a*_1_, *a*_2_, *a*_3_, and *a*_4_ in Eqs. **3** and **4** are estimated by performing 155 simulations of the full governing equations, spanning a wide range of values in the dimensionless groups to ensure proper coverage of the parameter space (*Materials and Methods*). Fig. 2*A* shows good agreement between the model Eq. **3** and simulations across four orders of magnitude in the slip velocity, for *a*_1_ ≈ 0.345, *a*_2_ ≈ 0.275, *a*_3_ ≈ 5.581, and *a*_4_ ≈ 3.922, which are values of order one as expected for scaling coefficients. The simulations also corroborate the assumption *γ*(*x*) ≈ ⟨*γ*⟩, as discussed in *SI Appendix*, *Finite-Element Simulations*.

Equipped with a 3D theory and a set of numerical simulation results, we aim to identify realistic combinations of the 10 dimensionless parameters that maximize the drag reduction of SHSs. Fig. 2 *B* and *C* illustrate how *λ*_*e*_ and *u*_*Ic*_ change with the spanwise gas fraction *ϕ*_*z*_, for a fixed bulk concentration ${\hat{c}}_{0}=3\cdot 10^{-4}\;\mathrm{mol}\;m^{-3}$ and $\hat{g}=1\;\mathrm{mm}$, and for several values of surfactant solubility, in a microchannel with $\hat{h}=60\;\mu m$. Slip is maximized for surfactant with higher solubility, corresponding to lower ${\hat{\kappa }}_{a}/{\hat{\kappa }}_{d}$. Incidentally, the results depend weakly on ${\hat{\kappa }}_{a}$ or ${\hat{\kappa }}_{d}$ individually; although these parameters appear separately in Eqs. 2 and **4** through *Bi* and *Da*, *Da* is large if $\hat{h}$ is larger than a few micrometer, such that the term *Bi*/(1 + *δ* *Da*)≈*Bi*/(*δDa*), which is a function of ${\hat{\kappa }}_{a}/{\hat{\kappa }}_{d}$ (*SI Appendix*, *Discussion of the Mobilization Length*).

Since the chemical properties of naturally occurring surfactants are virtually impossible to control in practice (32), we focus on geometrical parameters. We observe that *ϕ*_*z*_ has a negligible impact on surfactant effects, even as *ϕ*_*z*_ → 1. Mathematically, *ϕ*_*z*_ affects Eq. **3** only through the surfactant-independent term *u*_*Ic*_^clean^(*P*, *ϕ*_*z*_, *ϕ*_*x*_), which is at most of order one. Eqs. **3** and **4** reveal that slip is maximized by increasing the grating length *g*, which progressively overcomes surfactant effects, undergoing a transition of the form *u*_*Ic*_ ∼ *g*^2^ and ultimately approaching the asymptotes *u*_*Ic*_ → *u*_*Ic*_^clean^, *λ*_*e*_ → *λ*_*e*_^clean^. This transition is challenging to simulate due to the large computational cost of long domains needed at large *g*.

## Experiments Demonstrate Effect of Grating Length

**Fig. 3.**
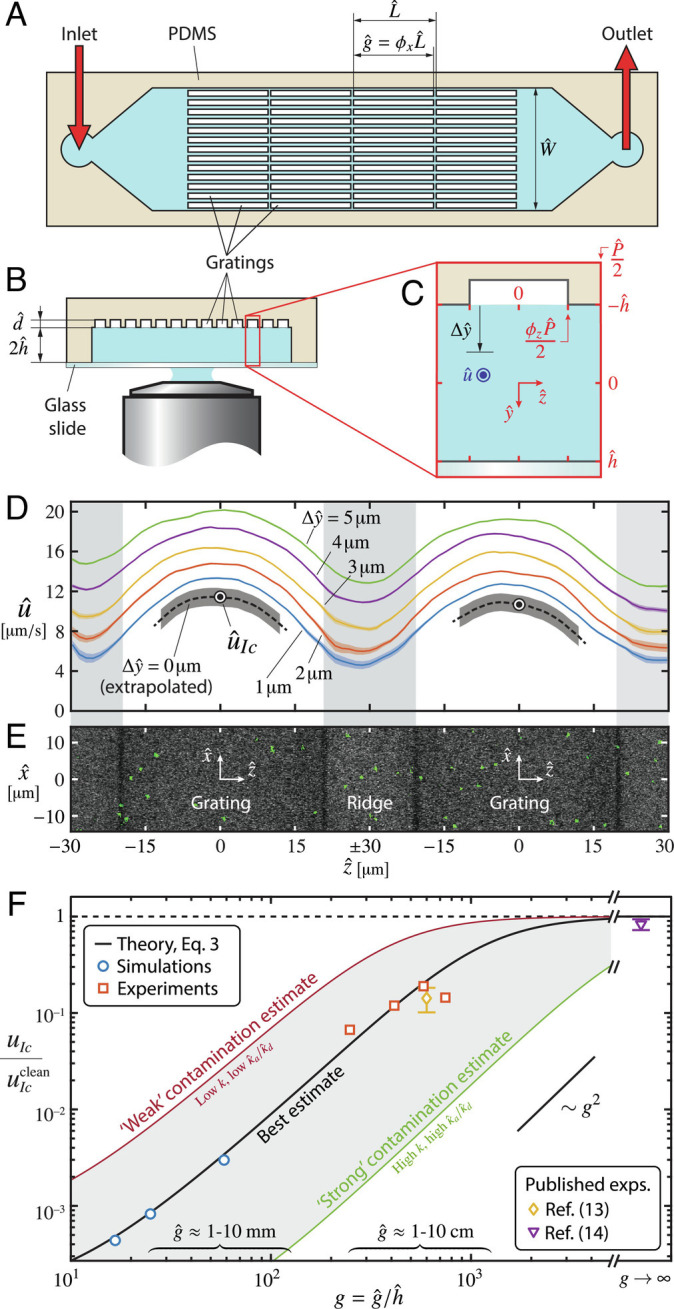
(*A*) Top view and (*B*) Cross-section of the microfluidic channels used in the experiments. (*C*) Cross-section of a unit cell, as defined in Fig. 1*B* (note the inverted microscope setup). The distance from the interface is $\Delta \hat{y}$. (*D*) Example of velocity profiles at different distances from the interface, for a grating length $\hat{g}=45\;$mm. The dashed line denotes the linearly extrapolated slip velocity, whereas the shadings show standard error (details in *SI Appendix*, *Experimental Methods*). The bull’s eyes mark the centerline slip velocity. (*E*) Micrograph of the gratings shown in (*D*), with μ-PIV particles appearing in green. (*F*) Ratio between the actual centerline slip velocity and the surfactant-free value (i.e., “clean”), $u_{\mathrm{Ic}}/u_{\mathrm{Ic}}^{\;\text{clean}}={\hat{u}}_{\mathrm{Ic}}/{{\hat{u}}_{\mathrm{Ic}}}^{\mathrm{clean}}$, from our theory (**3**), simulations, and experiments, as well as prior experiments of refs. (13) and (14). Ref. (14) used an annular grating in a rheometer, with an effectively infinite groove length. The theoretical prediction from our model **3** and the simulations use a best estimate of the unknown surfactant parameters (*SI Appendix*, *Estimate of Surfactant Parameters*), with the shaded region denoting a range of plausible levels of contamination within our estimates. The uncertainty in the present experimental data is smaller than the size of the symbols.

To acquire data at large *g* and test the prediction of a slip transition, we build microfluidic devices using polydimethylsiloxane (PDMS), as shown in Fig. 3 *A* and *B* (*Materials and Methods*). The channel upper wall consists of streamwise gratings of pitch $\hat{P}=60\;\mu \;\text{m}$ and spanwise gas fraction *ϕ*_*z*_ = 2/3. The channel half-height is $\hat{h}=60\;\mu \;\text{m}$, and the depth of the grating trenches is $\hat{d}=25\;\mu \;\text{m}$, enough to ensure a stable plastron throughout each experiment. We test gratings with $\hat{g}=15$, 25, 35  and  45 mm, separated in the streamwise direction by solid ridges of length $\hat{L}-\hat{g}=20\;\mu m$. We employ a confocal microscope and microparticle image velocimetry (μ-PIV) in a setup similar to the one in ref. (13). A syringe pump provides a constant flow rate ${\hat{Q}}_{\;\text{TOT}}=1.152\;\mu L\;\min^{-1}$. We use deionized water without any additives, since it has been established that the unavoidable amounts of surfactant naturally present in similar microfluidic settings are sufficient to induce significant stresses at the plastron (12–14). The μ-PIV beads used to seed the liquid (Fig. 3*E*) are thoroughly prewashed to remove their added surfactant (16), and we also follow a cleaning protocol for the syringes and tubing (*Materials and Methods*).

The flow velocity is measured over two adjacent gratings, at several distances $\Delta \hat{y}$ from the interface, as defined in Fig. 3*C*. Examples of velocity profiles are displayed in Fig. 3*D*, for $\hat{g}=45\;\mathrm{mm}$. The flow over the solid ridges is consistent with the no-slip condition at the wall, whereas velocity increases noticeably over the gratings. These vertically spaced profiles around the grating centerline are extrapolated to obtain the slip velocity at the interface, shown by the black dashed lines in Fig. 3*D*; the centerline slip velocity is marked by the bull’s eyes in the figure. The measured slip velocities ${\hat{u}}_{\mathrm{Ic}}$ are only 6 to 10% of the values ${\hat{u}}_{\mathrm{Ic}}^{\mathrm{clean}}$ predicted by surfactant-free theories (Fig. 3*F*), consistently with prior experiments (10–14).

Comparing quantitatively these experimental measurements to the predictions from our model requires assumptions on the type and amount of surfactant present in the channel. Although some parameter values are known and others can be accurately estimated, the normalized surfactant concentration *k* and the kinetic rate adsorption and desorption constants ${\hat{\kappa }}_{a}$ and ${\hat{\kappa }}_{d}$ can vary across a broad range. Nevertheless, it is possible to combine our model for the slip velocity Eq. **3** with previous experimental results (13) to obtain an estimate, as described in detail in *SI Appendix*, *Estimate of Surfactant Parameters*. We find approximate ranges for the normalized concentration 7.3 ⋅ 10^−3^ ≲ *k* ≲ 1 ⋅ 10^−1^ and for the ratio of constants $7.1\cdot 10^{1}\;m^{3}\;{\mathrm{mol}}^{-1}≲{\hat{\kappa }}_{a}/{\hat{\kappa }}_{d}≲1.8\cdot 10^{3}\;m^{3}\;{\mathrm{mol}}^{-1}$.

Choosing the mid-range values *k* = 3.6 ⋅ 10^−2^ and ${\hat{\kappa }}_{a}/{\hat{\kappa }}_{d}=1.2\cdot 10^{2}\;m^{3}\;{\mathrm{mol}}^{-1}$, our predictions of the slip velocity show good agreement with our experimental data and with previous studies (13, 14), as illustrated in Fig. 3*F*. At small *g*, measuring the small slip velocity with high precision is challenging; we performed finite-element simulations with the same surfactant properties as in the experiments, shown by the blue circles in Fig. 3*F*. These simulations are restricted to *g* <  60 (approximately 3.5 mm in practice), as computational cost increases with *g*. Simulations and experiments agree with Eq. **3**, showing increased slip consistent with the theoretical prediction as *g* increases and confirming the key impact of this geometric parameter in controlling drag reduction. Furthermore, all our measurements are consistent with the range of *k* and ${\hat{\kappa }}_{a}/{\hat{\kappa }}_{d}$ that we estimated from previous experiments in a different laboratory (13).

**Fig. 4.**
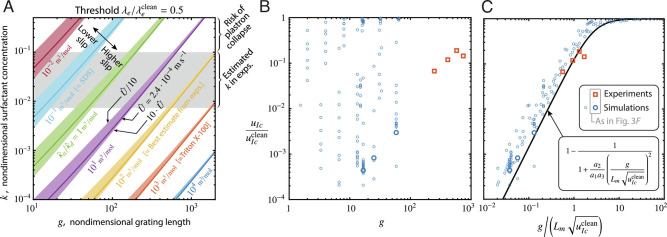
(*A*) Lines (from our model) showing the normalized surfactant concentration *k* as a function of grating length *g*, that yields a slip length that is 50% of the ideal, “clean” value. Colors denote different surfactant solubilities, expressed by the ratio of the adsorption and desorption constants ${\hat{\kappa }}_{a}/{\hat{\kappa }}_{d}$. Shaded bands show the weak effect of changing the flow velocity $\hat{U}$ across two orders of magnitude. (*B*) Experimental and numerical data for the slip velocity *u*_*Ic*_, plotted against *g*, as one varies surfactant properties, flow velocity, and SHS geometry, together characterized by ten dimensionless groups (including *g*). (*C*) Normalizing *g* by the mobilization length *L*_*m*_ approximately collapses the same data onto a single curve, governed by only one dimensionless group. Here, the factor ${\left(u_{\mathrm{Ic}}^{\mathrm{clean}}\right)}^{1/2}$ helps collapse data across a range of gas fractions, which give a wide range of values for $u_{\mathrm{Ic}}^{\mathrm{clean}}$. In practice, the normalized clean interface velocity $u_{\mathrm{Ic}}^{\mathrm{clean}}$ is usually of *O*(1), so this factor could be omitted in the normalization for *g*.

To assess practical implications for general microfluidic flows, we plot in Fig. 4*A* the combinations of *g* and *k* at which the slip length reaches 50% of the surfactant-free value (i.e., *λ*_*e*_/*λ*_*e*_^clean^ = 0.5, plotted with solid lines), for surfactant solubility values ${\hat{\kappa }}_{a}/{\hat{\kappa }}_{d}$ (plotted with different colors) and a range of representative velocities $\hat{U}$ (shown by shaded colored bands around each line) found in small-scale applications. The gray horizontal band shows the range 7 ⋅ 10^−3^ ≲ *k* ≲ 0.1 estimated for microfluidic experiments. At larger values *k* >  0.1, the risk of plastron collapse increases significantly due to capillary effects. Increasing or reducing the velocity $\hat{U}$ by an order of magnitude has a relatively weak effect on the slip, as shown by the narrow bands around each line in Fig. 4*A*. In addition, Fig. 4*A* shows that varying ${\hat{\kappa }}_{a}/{\hat{\kappa }}_{d}$ leads to uniformly shifted contours, which remain approximately parallel. Together with the weak dependence on velocity, these results suggest that the 50% threshold for slip could be expressed through a simpler underlying criterion.

## Single Lengthscale Predicts Interface Mobility

Fig. 3*F* shows that surfactant impairment is strongly dependent on the grating length *g*. We therefore define the mobilization length *L*_*m*_ as the value of *g* that gives significant slip, such that the local slip length on the interface is *λ*_*I*_(*L*_*m*_)∼1 (or in dimensional terms, ${\hat{\lambda }}_{I}\left({\hat{L}}_{m}/\hat{h}\right)∼\hat{h}$). We seek a scaling for *λ*_*I*_ in terms of *g*, starting from the definition of *λ*_*I*_ and using Eq. **2d**,

[5] $$\begin{array}{c} \lambda_{I}=\frac{u_{I}}{\gamma }∼\frac{u_{I}}{kMa\frac{\Delta \Gamma }{g}}. \end{array}$$

Note that *x* is normalized by $\hat{L}$, whereas *g* is normalized by $\hat{h}$, such that gradients along the interface occur over an *x* length scale $\hat{g}/\hat{L}∼\varepsilon g$. Here, *ΔΓ* is the order of magnitude of the change in *Γ* along the interface, illustrated in Fig. 1*D*. More specifically, we assume that *c*_*I*_ ranges from 1 − *Δc*_*I*_ to 1 + *Δc*_*I*_, and similarly, *Γ* is between 1 − *ΔΓ* and 1 + *ΔΓ*, such that at some point near the middle of the interface, *Γ* ∼ *c* ∼ 1. Scaling Eq. **2c**,

[6] $$\begin{array}{c} -\frac{\Delta c_{I}}{\delta }∼Da\;\left(\Delta c_{I}-\Delta \Gamma \right),\;\;\text{i.e.}\;\Delta \Gamma ∼\Delta c_{I}(\frac{1}{\delta \;Da}+1). \end{array}$$

We find *Δc*_*I*_ by scaling Eq. **2b**, which represents a balance between advection, which acts to establish a surfactant gradient, and diffusion and kinetics, which act to extinguish the gradient. We consider changes along the *x*-direction, between the edge of the interface (where *u* = 0) and a point near the middle (where *u* ∼ *u*_*I*_ and *Γ* ∼ 1). Then, ∂_*x*_(*uΓ*)∼*u*_*I*_/(*εg*) and ∂_*xx*_*Γ* ∼ *ΔΓ*/(*εg*)^2^. Writing the kinetic flux *Bi*(*c*_*I*_ − *Γ*)=(*Bi*/*Da*) ∂_*y*_*c*|_*I*_ and using Eq. **6** to eliminate *ΔΓ*, Eq. **2b** yields

[7] $$\begin{array}{c} \frac{u_{I}}{g}∼(Bi+\frac{1+\delta \mathrm{Da}}{Pe_{I}g^{2}})\frac{\Delta c_{I}}{\delta \mathrm{Da}}. \end{array}$$

The second term in the parenthesis on the right-hand side is negligible if diffusion is weak compared to kinetics, such that *g*^2^ ≫ (1 + *δDa*)/(*Bi* *Pe*_*I*_). Note that *δ* ∼ 1 unless the gratings are very short, and recall that *Da* is large if $\hat{h}$ is larger than a few micrometers, such that (1 + *δ* *Da*)∼*Da*. In dimensional form, neglecting diffusion in Eq. **7** then requires ${\hat{g}}^{2}\gg {\left({\hat{L}}_{d}^{\;\text{mod}}\right)}^{2}$, where


[8] $$\begin{array}{c} {\hat{L}}_{d}^{\mathrm{mod}}:={\left(\hat{h}{\hat{L}}_{d}\frac{{\hat{D}}_{I}}{\hat{D}}\right)}^{1/2}, \end{array}$$

and ${\hat{L}}_{d}={\hat{\kappa }}_{a}{\hat{\Gamma }}_{m}/{\hat{\kappa }}_{d}$ is the depletion length, which arises commonly in models of soluble surfactant (19). We define ${\hat{L}}_{d}^{\;\text{mod}}$ as a modified depletion length, representing the grating length above which interface diffusion is small compared to kinetics. Omitting the diffusive term in (**7**), and combining with (**5**) and (**6**) to eliminate *ΔΓ* and *Δc*_*I*_, we find

[9] $$\begin{array}{c} \lambda_{I}∼\frac{g^{2}\;Bi}{k\;Ma\;Da}. \end{array}$$

This scaling shows concisely that *λ*_*I*_ depends on *g*^2^, consistently with Eq. **4** and with Fig. 3*F*. If *λ*_*I*_ ∼ 1 when *g* ∼ *L*_*m*_, Eq. **9** defines the mobilization length; in dimensional form, we find

[10] $$\begin{array}{c} {\hat{L}}_{m}:=\hat{h}\frac{{\hat{\kappa }}_{a}{\hat{\Gamma }}_{m}}{{\hat{\kappa }}_{d}}{\left(\frac{n_{s}\hat{R}\hat{T}{\hat{c}}_{0}}{\hat{\mu }\hat{D}}\right)}^{1/2}. \end{array}$$

Marangoni stresses become negligible if ${\hat{g}}^{2}\gg {{\hat{L}}_{m}}^{2}$ as transport between the bulk and the interface suppresses the surfactant gradient that would otherwise be established by advection. For our experiments (*SI Appendix*, Table SI), Eq. **10** predicts ${\hat{L}}_{m}\approx 4.3\;$cm, indicating that gratings must be at least several centimeters long to minimize Marangoni stresses, consistently with Fig. 3*F*. Remarkably, ${\hat{L}}_{m}$ depends linearly on ${\hat{\kappa }}_{a}/{\hat{\kappa }}_{d}$ but only on the square root of ${\hat{c}}_{0}$, showing higher sensitivity to the type of surfactant than to its concentration.

For small-scale applications, and using properties of known surfactants, we find that ${\hat{L}}_{d}^{\;\text{mod}}$ is much smaller than ${\hat{L}}_{m}$; for example, ${\hat{L}}_{d}^{\;\text{mod}}\approx 170\;\mu m$ in our experiments (*SI Appendix*, Table SI and *SI Appendix*, *Discussion of the Mobilization Length*). Therefore, the mobilization length ${\hat{L}}_{m}$ is the key length scale that determines the slip of a given SHS.

Our analysis suggests that it should be possible to write simplified expressions for *u*_*Ic*_ and *λ*_*e*_ in terms of *g*/*L*_*m*_. With the approximation (1 + *δDa*)≈*Da* (as discussed above) and assuming ${\hat{L}}_{m}\gg {\hat{L}}_{d}^{\;\text{mod}}$ (such that the terms 1/*Pe*_*I*_ are negligible), Eqs. **3** and **4** simplify to the one-parameter curves

[11] $$\begin{array}{cc} \frac{u_{\mathrm{Ic}}}{u_{\mathrm{Ic}}^{\mathrm{clean}}} & =1-\frac{1}{1+\frac{a_{2}}{a_{1}a_{3}}{\left(\frac{g}{L_{m}\sqrt{u_{\mathrm{Ic}}^{\mathrm{clean}}}}\right)}^{2}}, \end{array}$$

[12] $$\begin{array}{cc} \frac{\lambda_{e}}{\lambda_{e}^{\mathrm{clean}}} & =1-\frac{1}{1+\frac{a_{2}}{a_{1}a_{3}}{\left(\frac{g}{L_{m}\sqrt{u_{\mathrm{Ic}}^{\mathrm{clean}}(1+\frac{\lambda_{e}^{\mathrm{clean}}}{2})}}\right)}^{2}}. \end{array}$$

We reexamine the 155 simulations shown in Fig. 2*A* and set aside 24 cases that are not achievable in reality (e.g. involving unphysically small diffusivities). Fig. 4*B* shows the relative slip *u*_*Ic*_/*u*_*Ic*_^clean^ versus *g* for the remaining 131 simulations and for our experiments. These data span different surfactant properties, grating geometries and flow velocities, and exhibit large scatter, illustrating how *g* alone is insufficient to predict slip. However, when *g* is normalized by the mobilization length *L*_*m*_, the same data collapse near the one-parameter curve given by Eq. **11**, as shown in Fig. 4*C*.

## Discussion and Outlook

Regarding the surfactant type inherent to our experiments, Eqs. **10** and **11** suggest a surfactant with large ${\hat{\kappa }}_{a}/{\hat{\kappa }}_{d}$, implying low solubility. This is consistent with previous findings that PDMS used in microfluidic channels (including our experiments) releases uncrosslinked oligomer chains (36–38), which are surface active (31, 32), and which have also been detected in solution (30, 33). The mass fractions reported in ref. (33), in combination with the oligomer chain lengths found in ref. (30), lead to concentrations ${\hat{c}}_{0}∼O\left(10^{-4}-10^{-2}\right)\;\mathrm{mol}\;m^{-3}$, compatible with our estimates. Incidentally, in other contexts, PDMS has sometimes been approximately modeled as insoluble (39, 40); under this assumption, our theory yields *u*_*Ic*_ = *u*_*Ic*_^clean^/(1 + *a*_ins_ *Ma*_ins_ *u*_*Ic*_^clean^) (*SI Appendix*, *Scaling Theory for Surfactant Transport*), where *a*_ins_ is a scaling coefficient, $Ma_{\mathrm{ins}}=n_{s}\hat{R}\hat{T}{\hat{\Gamma }}_{0}\hat{h}/\left(\hat{\mu }{\hat{D}}_{I}\right)$ is a Marangoni number, and ${\hat{\Gamma }}_{0}$ is the average interfacial surfactant concentration. Note that this expression for *u*_*Ic*_ does not depend on *g*, inconsistently with the experimental results in Fig. 3*F*. This highlights the importance of including solubility in models of surfactant dynamics on SHSs.

The results described here provide insights into the slip and drag of superhydrophobic surfaces in realistic conditions. Our theory for slender, finite gratings (which are widely used) enables comparisons with experiments, where inherent surfactants must be accounted for. The hydrodynamic component of the model from Eq. **1** can also quantify SHS performance in the presence of general, nonuniform shear stresses at the air–water interface, thus circumventing the need for computationally expensive simulations. In addition, we have shown that a single mobilization length scale arising from the theory can serve as a guide in the design of SHS textures that mitigate surfactant effects. This laminar theory is also a stepping stone toward predicting surfactant effects in turbulent flow. As a first approximation, the mobilization length could be estimated by replacing the channel half-height $\hat{h}$ in Eq. **10** with the thickness of the viscous sublayer. Finally, since ${\hat{L}}_{m}$ depends primarily on surfactant properties and on the shear length scale $\hat{h}$, we may also expect that the concept of mobilization length, derived here for streamwise gratings, could qualitatively apply to other SHS textures.

## Materials and Methods

### Finite-Element Simulations.

We solved the full governing equations and boundary conditions (in dimensional form, detailed in *SI Appendix*, *Governing Equations*) in three dimensions using COMSOL Multiphysics 5.5. We performed a total of 155 simulations using different grating geometries, flow velocities, and surfactant properties in order to span a large portion of the parameter space characterized by the ten dimensionless numbers of the problem. The domain was one half of the SHS unit cell depicted in Fig. 1*B*, with $\hat{z}$ between $\hat{z}=0$ and $\hat{z}=\hat{P}/2$ due to the spanwise symmetry of the solution. The volume is meshed with tetrahedral elements, with the finest ones (with a minimum element size of 1.5 ⋅ 10^−9^ m) around the upstream and downstream edges of the interface $\hat{x}=\pm \phi_{x}\hat{L}/2$ (*SI Appendix*, *Finite-Element Simulations*). We used the Creeping Flow module for the flow field, the Dilute Species Transport module for the transport of a bulk surfactant, and the transport of an interfacial surfactant was implemented through a General Form Boundary PDE. The Marangoni boundary conditions were enforced through a Weak Contribution constraint, as was the condition that fixed the mean bulk concentration to be ${\hat{c}}_{0}$. The system of nonlinear equations was solved through a Newton-type iterative method using the PARDISO direct solver for the linear system at each iteration. All simulations satisfied a relative tolerance for convergence of 1 ⋅ 10^−5^. We used linear elements for the pressure, bulk concentration and interfacial concentration, and either linear or quadratic elements for the velocity field, depending on the computational demands of each simulation.

### Microchannel Fabrication.

Microfluidic channels with an array of parallel SHS gratings on their ceiling (Fig. 3 *A* and *B*) were built by casting PDMS (Sylgard 184) over a mold obtained by two-layer photolithography. The photoresist used was SU-8 (Microchem SU-8 3025 and Microchem SU-8 3050). The chips were bonded to 0.1 mm-thick glass coverslips (Bellco Glass 1916-25075) through untreated adhesion. Every coverslip was washed with isopropyl alcohol, then with 18 M*Ω* cm DI water, and finally dried with nitrogen before the microfluidic chip was attached. The static contact angle of water droplets was measured to be higher than 100° over samples of smooth PDMS, and to increase further over samples of textured PDMS. This is consistent with previous measurements for untreated PDMS (41) and demonstrates the superhydrophobicity of the substrate. The total width of the microfluidic channel was set to $\hat{W}=2\;\mathrm{mm}$ (Fig. 3*A*) to ensure an approximately periodic flow in $\hat{z}$ over the gratings far way from lateral walls (given that $\hat{W}\gg \hat{h}$).

### Experimental Setup.

A glass syringe (Hamilton Gastight) was filled with particle-seeded DI water, which was driven through the microchannels using a syringe pump (KD Legato 111). We used the barrel of a plastic syringe (BD Luer-Lok) as an outlet reservoir open to the room, to impose atmospheric pressure at the end of the circuit. The height of this reservoir was adjusted with a vertical translation stage (Thorlabs VAP10) at the beginning of each experiment to ensure that the plastron at each grating remained approximately flat, by controlling the average pressure in the microchannel. The microchannel was connected to the syringe and reservoir through plastic tubing (Tygon S3). All circuit elements were thoroughly prewashed with 18 M*Ω* cm DI water, following a protocol described in *SI Appendix*, *Experimental Methods*.

### Confocal Microscopy.

The tracer particles (ThermoFisher FluoSpheres carboxylate 0.5 μm diameter) were washed using a centrifuge (Eppendorf 5418) to separate them from the buffer solution, which was then discarded and replenished with 18 M*Ω* cm DI water. This process was repeated three times before each experiment to essentially eliminate surfactant contamination from the particle solution. The flow was observed with a confocal microscope (Leica SP8 Resonant Scanning), using a 40× water objective (as in Fig. 3*B*). The microfluidic device was enclosed in a stage top chamber (Okolab H101-K-FRAME) with a controlled temperature set to $\hat{T}=296\;K$. Using the bright field imaging of the microscope (Fig. 3*E*), we focused on two adjacent gratings. We avoided imaging the five gratings closest to each lateral side wall of the channel to prevent effects related to the loss of periodicity. The fluorescence imaging of the microscope (superimposed on the image in Fig. 3*E*) revealed the positions of the tracer beads in each snapshot, which we obtained at a rate of between 20 and 28 frames per second. All the data were taken at the center of the grating in the streamwise direction (i.e. $\hat{x}\approx 0$) and at several distinct $\hat{y}$-planes close to the interface (Fig. 3*D*).

### Image Analysis and Micro-PIV.

The μ-PIV analysis was performed with the open-source MATLAB toolbox PIVlab (42), using an acquisition window of approximately 125 μm × 125 μm. The velocity field obtained for a given window was averaged in time and along the streamwise $\hat{x}$ direction to obtain the spanwise velocity profiles depicted in Fig. 3*D*, for different distances away from the interface. To extract the centerline slip velocity ${\hat{u}}_{\mathrm{Ic}}$ (at $\hat{y}=-\hat{h}$ and $\hat{z}=0$), we performed a linear least-squares fit using data from between three to five $\hat{y}$-planes. We only used data in a neighborhood of the grating center $\hat{z}=0$ (Fig. 3*D*), since velocity profiles at $\hat{y}=-\hat{h}$ were not smooth due to the transitions between the interfaces and the solid ridges at $\hat{z}=\pm \phi_{z}\hat{P}/2$. The uncertainty for ${\hat{u}}_{\mathrm{Ic}}$ was calculated by accounting for how uncertainties in the velocity measurements and in the $\hat{y}$-coordinate of the interface (±1 μm) propagated through the fitting procedure.

## Supplementary Material

Appendix 01 (PDF)

Dataset S01 (XLSX)

## Data Availability

All study data are included in the article and/or *SI Appendix*.
