# Supplementary material for: A single parameter can predict surfactant impairment of superhydrophobic drag reduction
Source: Proc Natl Acad Sci U S A. 2023 Jan 12;120(3):e2211092120. doi: 10.1073/pnas.2211092120 (PMC9934013; doi:10.1073/pnas.2211092120)
Supplement: Supplementary file 1 — Appendix 01 (PDF) [file pnas.2211092120.sapp.pdf]

Supporting Information for

A single parameter can predict surfactant impairment of superhydrophobic drag reduction

F. Temprano-Coleto, S. M. Smith, F. J. Peaudecerf, J. R. Landel, F. Gibou, and P. Luzzatto-Fegiz

Paolo Luzzatto-Fegiz.
E-mail: pfegiz@ucsb.edu

This PDF file includes:

Supporting text
Figs. S1 to S2
Tables S1 to S2
Legend for Dataset S1
SI References

Other supporting materials for this manuscript include the following:

Dataset S1

Supporting Information Text

Governing equations

We consider a steady fluid flow at low Reynolds number, within the unit cell depicted in Fig. 1A, B. The three-dimensional velocity field is denoted by $\hat{\boldsymbol{u}} = \hat{u}\,\boldsymbol{e}_x + \hat{v}\,\boldsymbol{e}_y + \hat{w}\,\boldsymbol{e}_z$, where $\boldsymbol{e}_x$, $\boldsymbol{e}_y$ and $\boldsymbol{e}_z$ are unit vectors in the streamwise, wall-normal and spanwise directions (see Fig. 1C). The scalar fields $\hat{p}$ and $\hat{c}$ represent the pressure and the bulk surfactant concentration, respectively. The governing equations describing the conservation of mass, momentum, and surfactant in the bulk fluid are, in dimensional form,

$$\frac{\partial \hat{u}}{\partial \hat{x}} + \frac{\partial \hat{v}}{\partial \hat{y}} + \frac{\partial \hat{w}}{\partial \hat{z}} = 0, \qquad \text{[S1a]}$$

$$\hat{\mu}\left(\frac{\partial^2 \hat{u}}{\partial \hat{x}^2} + \frac{\partial^2 \hat{u}}{\partial \hat{y}^2} + \frac{\partial^2 \hat{u}}{\partial \hat{z}^2}\right) = \frac{\partial \hat{p}}{\partial \hat{x}}, \qquad \text{[S1b]}$$

$$\hat{\mu}\left(\frac{\partial^2 \hat{v}}{\partial \hat{x}^2} + \frac{\partial^2 \hat{v}}{\partial \hat{y}^2} + \frac{\partial^2 \hat{v}}{\partial \hat{z}^2}\right) = \frac{\partial \hat{p}}{\partial \hat{y}}, \qquad \text{[S1c]}$$

$$\hat{\mu}\left(\frac{\partial^2 \hat{w}}{\partial \hat{x}^2} + \frac{\partial^2 \hat{w}}{\partial \hat{y}^2} + \frac{\partial^2 \hat{w}}{\partial \hat{z}^2}\right) = \frac{\partial \hat{p}}{\partial \hat{z}}, \qquad \text{[S1d]}$$

$$\hat{u}\frac{\partial \hat{c}}{\partial \hat{x}} + \hat{v}\frac{\partial \hat{c}}{\partial \hat{y}} + \hat{w}\frac{\partial \hat{c}}{\partial \hat{z}} = \hat{D}\left(\frac{\partial^2 \hat{c}}{\partial \hat{x}^2} + \frac{\partial^2 \hat{c}}{\partial \hat{y}^2} + \frac{\partial^2 \hat{c}}{\partial \hat{z}^2}\right). \qquad \text{[S1e]}$$

At the interface, the interfacial surfactant concentration $\hat{\Gamma}$ follows a conservation law. An adsorption–desorption flux couples $\hat{\Gamma}$ to the bulk concentration. Marangoni boundary conditions link the interfacial shear stress to the concentration gradient. The corresponding equations, defined only at the air–water interface, read

$$\frac{\partial(\hat{u}_I\hat{\Gamma})}{\partial \hat{x}} + \frac{\partial(\hat{w}_I\hat{\Gamma})}{\partial \hat{z}} = \hat{D}_I\left(\frac{\partial^2 \hat{\Gamma}}{\partial \hat{x}^2} + \frac{\partial^2 \hat{\Gamma}}{\partial \hat{z}^2}\right) + \hat{\mathcal{S}}(\hat{c}_I, \hat{\Gamma}), \qquad \text{[S1f]}$$

$$\hat{D}\left.\frac{\partial \hat{c}}{\partial \hat{y}}\right|_I = \hat{\mathcal{S}}(\hat{c}_I, \hat{\Gamma}), \qquad \text{[S1g]}$$

$$\hat{\mu}\left.\frac{\partial \hat{u}}{\partial \hat{y}}\right|_I = \hat{\mathcal{N}}(\hat{\Gamma})\frac{\partial \hat{\Gamma}}{\partial \hat{x}}, \qquad \text{[S1h]}$$

$$\hat{\mu}\left.\frac{\partial \hat{w}}{\partial \hat{y}}\right|_I = \hat{\mathcal{N}}(\hat{\Gamma})\frac{\partial \hat{\Gamma}}{\partial \hat{z}}, \qquad \text{[S1i]}$$

where $\hat{\mathcal{N}}(\hat{\Gamma})$ is a possibly nonlinear term quantifying the dependence of the surface tension with $\hat{\Gamma}$, and depends on the specific model of equilibrium isotherm chosen (1). The term $\hat{\mathcal{S}}(\hat{c}_I, \hat{\Gamma})$ represents the adsorption-desorption kinetics, and must be compatible with the choice of isotherm. Here, we use a model derived from the Frumkin isotherm (2, 3), which leads to

$$\hat{\mathcal{S}}(\hat{c}_I, \hat{\Gamma}) = \hat{\kappa}_a\hat{c}_I(\hat{\Gamma}_m - \hat{\Gamma}) - \hat{\kappa}_d\hat{\Gamma}e^{A\hat{\Gamma}/\hat{\Gamma}_m}, \qquad \text{[S1j]}$$

$$\hat{\mathcal{N}}(\hat{\Gamma}) = n_s\hat{R}\hat{T}\left(\frac{\hat{\Gamma}_m}{\hat{\Gamma}_m - \hat{\Gamma}} + A\frac{\hat{\Gamma}}{\hat{\Gamma}_m}\right). \qquad \text{[S1k]}$$

The above equations are complemented with the imposition of a mean background level of bulk concentration

$$\frac{1}{2\hat{h}\hat{P}\hat{L}}\int_{-\hat{P}/2}^{\hat{P}/2}\int_{-\hat{h}}^{\hat{h}}\int_{-\hat{L}/2}^{\hat{L}/2}\hat{c}\,\mathrm{d}\hat{x}\,\mathrm{d}\hat{y}\,\mathrm{d}\hat{z} = \hat{c}_0, \qquad \text{[S1l]}$$

as well as with streamwise and spanwise periodicity conditions for variables defined in the bulk fluid,

$$\hat{\boldsymbol{u}}(\hat{\boldsymbol{x}}) = \hat{\boldsymbol{u}}(\hat{\boldsymbol{x}} + \alpha\hat{L}\boldsymbol{e}_x + \beta\hat{P}\boldsymbol{e}_z) \text{ for any integers } \alpha,\ \beta, \qquad \text{[S1m]}$$

$$\hat{c}(\hat{\boldsymbol{x}}) = \hat{c}(\hat{\boldsymbol{x}} + \alpha\hat{L}\boldsymbol{e}_x + \beta\hat{P}\boldsymbol{e}_z) \text{ for any integers } \alpha,\ \beta, \qquad \text{[S1n]}$$

which in the case of the pressure also includes a mean pressure drop such that

$$\hat{p}(\hat{\boldsymbol{x}}) = \hat{p}(\hat{\boldsymbol{x}} + \alpha\hat{L}\boldsymbol{e}_x + \beta\hat{P}\boldsymbol{e}_z) + \alpha\hat{G}\hat{L} \text{ for any integers } \alpha,\ \beta, \qquad \text{[S1o]}$$

and where $\hat{\boldsymbol{x}} = \hat{x}\,\boldsymbol{e}_x + \hat{y}\,\boldsymbol{e}_y + \hat{z}\,\boldsymbol{e}_z$ is the position vector. The remaining equations are the boundary conditions

$$\hat{\boldsymbol{u}} = \boldsymbol{0} \text{ on all solid surfaces (no slip and no penetration),} \qquad \text{[S1p]}$$

$$\hat{v} = 0 \text{ on the air–water interface (no penetration),} \qquad \text{[S1q]}$$

$$\frac{\partial \hat{c}}{\partial \hat{y}} = 0 \quad \text{on all solid surfaces (no flux)}, \tag{S1r}$$

$$\frac{\partial \hat{\Gamma}}{\partial \hat{x}} = 0 \quad \text{at } \hat{x} = \pm\phi_x \hat{L}/2 \text{ when } |\hat{z}| \leq \phi_z \hat{P}/2 \text{ (no flux)}, \tag{S1s}$$

$$\frac{\partial \hat{\Gamma}}{\partial \hat{z}} = 0 \quad \text{at } \hat{z} = \pm\phi_z \hat{P}/2 \text{ when } |\hat{x}| \leq \phi_x \hat{L}/2 \text{ (no flux)}. \tag{S1t}$$

We normalize Eqs. S1a-S1t following

$$\begin{gathered} x = \hat{x}/\hat{L}, \quad y = \hat{y}/\hat{h} = \hat{y}/(\varepsilon\hat{L}), \quad z = \hat{z}/\hat{h} = \hat{z}/(\varepsilon\hat{L}) \\ u = \hat{u}/\hat{U}, \quad v = \hat{v}/(\varepsilon\hat{U}), \quad w = \hat{w}/(\varepsilon\hat{U}), \quad p = \hat{p}/(\hat{G}\hat{L}) \\ c = \hat{c}/\hat{c}_0, \quad \Gamma = \hat{\Gamma}/\hat{\Gamma}_0, \end{gathered} \tag{S2}$$

where $\hat{U} = \hat{h}^2\hat{G}/\hat{\mu}$ and $\hat{\Gamma}_0 = \hat{\kappa}_a\hat{c}_0\hat{\Gamma}_m/\hat{\kappa}_d$ are the natural scales for the velocity and the interfacial surfactant. Applying this normalization to Eqs. S1a-S1k results in

$$\frac{\partial u}{\partial x} + \frac{\partial v}{\partial y} + \frac{\partial w}{\partial z} = 0, \tag{S3a}$$

$$\left(\varepsilon^2\frac{\partial^2 u}{\partial x^2} + \frac{\partial^2 u}{\partial y^2} + \frac{\partial^2 u}{\partial z^2}\right) = \frac{\partial p}{\partial x}, \tag{S3b}$$

$$\varepsilon^2\left(\varepsilon^2\frac{\partial^2 v}{\partial x^2} + \frac{\partial^2 v}{\partial y^2} + \frac{\partial^2 v}{\partial z^2}\right) = \frac{\partial p}{\partial y}, \tag{S3c}$$

$$\varepsilon^2\left(\varepsilon^2\frac{\partial^2 w}{\partial x^2} + \frac{\partial^2 w}{\partial y^2} + \frac{\partial^2 w}{\partial z^2}\right) = \frac{\partial p}{\partial z}, \tag{S3d}$$

$$u\frac{\partial c}{\partial x} + v\frac{\partial c}{\partial y} + w\frac{\partial c}{\partial z} = \frac{1}{\varepsilon Pe}\left(\varepsilon^2\frac{\partial^2 c}{\partial x^2} + \frac{\partial^2 c}{\partial y^2} + \frac{\partial^2 c}{\partial z^2}\right). \tag{S3e}$$

$$\frac{\partial(u_I\Gamma)}{\partial x} + \frac{\partial(w_I\Gamma)}{\partial z} = \frac{1}{\varepsilon Pe_I}\left(\varepsilon^2\frac{\partial^2 \Gamma}{\partial x^2} + \frac{\partial^2 \Gamma}{\partial z^2}\right) + \frac{Bi}{\varepsilon}\mathcal{S}(c_I,\Gamma), \tag{S3f}$$

$$\left.\frac{\partial c}{\partial y}\right|_I = Da\,\mathcal{S}(c_I,\Gamma), \tag{S3g}$$

$$\left.\frac{\partial u}{\partial y}\right|_I = \varepsilon k Ma\,\mathcal{N}(\Gamma)\frac{\partial \Gamma}{\partial x}, \tag{S3h}$$

$$\varepsilon^2\left.\frac{\partial w}{\partial y}\right|_I = \varepsilon k Ma\,\mathcal{N}(\Gamma)\frac{\partial \Gamma}{\partial z}, \tag{S3i}$$

$$\mathcal{S}(c_I,\Gamma) = c_I(1-k\Gamma) - \Gamma e^{kA\Gamma}, \tag{S3j}$$

$$\mathcal{N}(\Gamma) = \left(\frac{1}{1-k\Gamma} + kA\Gamma\right). \tag{S3k}$$

Problem parameters

The parameters appearing in Eqs. S1a-S3k are defined in Tables SI and SII, together with the values that they take in our experiments. Since, as explained in the main text, the surfactant type and concentration in the liquid are unknown, only an estimate can be obtained in some cases (see Section *Estimate of surfactant parameters* for details). We choose $g = \hat{g}/\hat{h}$, $P = \hat{P}/\hat{h}$, ϕ_x and ϕ_z as the four independent geometric parameters of the problem, noting that ε can then be obtained as $\varepsilon = \phi_x/g$. Tables SI and SII also include the values of the length scales $\hat{L}_d^{\mathrm{mod}}$ and $\hat{L}_m$, defined in Eqs. 10 and 9 of the main text.

Flow field derivation

Assumption of a spanwise constant interface shear stress. Note that, although $\varepsilon \ll 1$ and $k \ll 1$ in the conditions considered in our study (see Section *Scaling theory for surfactant transport*), the product $\varepsilon k Ma$ appearing in [S3h] and [S3i] is typically not small, since the Marangoni number is expected to be large, i.e. $Ma \gg 1$ (see estimates in Table SII) and the term $\mathcal{N}(\Gamma) \approx 1$ as long as k and $k|A|$ remain small. In fact, Eq. S3h implies that only when $\varepsilon k Ma \gtrsim 1$ the Marangoni stresses at the interface are non-negligible, as it is observed experimentally (4–7). Since $\varepsilon \ll 1$, it is possible to assume that $\varepsilon k Ma \gtrsim 1 \gg \varepsilon^2$, and in that case it follows from [S3i] that $\partial_z\Gamma \approx 0$ at leading order in ε. As detailed in the main text, the asymptotic expansion leading to [S3h] and [S3i] is singular, and thus the approximation $\partial_z\Gamma \approx 0$ is valid only in regions far from the upstream and downstream stagnation points, i.e. for $|x \pm \phi_x/2| \gg \varepsilon$. Indeed, our finite-element simulations of the full problem confirm that this approximation remains valid in all the regimes considered (as illustrated in Fig. S1b, showing the contours of Γ). The Marangoni shear $\gamma(x) = \partial_y u|_I$ is thus also assumed to be independent of z and only dependent on x, following [S3h].

Table SI. Parameters appearing in the dimensional Eqs. S1a–S2 and defining the flow geometry (Fig. 1), alongside with their values in the simulations and experiments. The symbol ‡ indicates quantities whose order of magnitude does not change appreciably across surfactants; the values are for sodium dodecyl sulfate (SDS), which is well characterized (2). The symbol † denotes values that have been estimated by combining our theory and the experimental results in (4) (see Section *Estimate of surfactant parameters* for details).

Quantity	Symbol	Units	Value (or best estimate) in experiments
Background bulk concentration	$\hat{c}_0$	$\mathrm{mol\,m^{-3}}$	$3 \cdot 10^{-4}$ †
Adsorption rate constant	$\hat{\kappa}_a$	$\mathrm{m^3\,mol^{-1}\,s^{-1}}$	$8.95 \cdot 10^{1}$ †
Desorption rate constant	$\hat{\kappa}_d$	$\mathrm{s^{-1}}$	$7.5 \cdot 10^{-1}$ †
Maximum packing concentration	$\hat{\Gamma}_m$	$\mathrm{mol\,m^{-2}}$	$3.9 \cdot 10^{-6}$ ‡
Bulk surfactant diffusivity	$\hat{D}$	$\mathrm{m^2\,s^{-1}}$	$7 \cdot 10^{-10}$ ‡
Interface surfactant diffusivity	$\hat{D}_I$	$\mathrm{m^2\,s^{-1}}$	$7 \cdot 10^{-10}$ ‡
Salinity parameter	n_s	-	2 ‡
Frumkin interaction coefficient	A	-	-2.4 ‡
Ideal gas constant	$\hat{R}$	$\mathrm{J\,mol^{-1}\,K^{-1}}$	8.314
Temperature	$\hat{T}$	K	296
Dynamic viscosity	$\hat{\mu}$	$\mathrm{kg\,m^{-1}\,s^{-1}}$	$8.9 \cdot 10^{-4}$
Velocity scale	$\hat{U}$	$\mathrm{m\,s^{-1}}$	$2.4 \cdot 10^{-4}$
Channel half height (see Fig. 1)	$\hat{h}$	m	$6 \cdot 10^{-5} \pm 3 \cdot 10^{-6}$
Pitch (see Fig. 1)	$\hat{P}$	m	$6 \cdot 10^{-5}$
Grating width (see Fig. 1)	$\phi_z \hat{P}$	m	$4 \cdot 10^{-5}$
Grating length (see Fig. 1)	$\hat{g}$	m	$(1.5, 2.5, 3.5, 4.5) \cdot 1 \cdot 10^{-2}$
Ridge size in x (see Fig. 1)	$\hat{L} - \hat{g}$	m	$2 \cdot 10^{-5}$
Depletion length (see Eq. 8)	$\hat{L}_d = \hat{\kappa}_a \hat{\Gamma}_m / \hat{\kappa}_d$	m	$4.7 \cdot 10^{-4}$
Modified depletion length (see Eq. 8)	$\hat{L}_d^{\text{mod}} = \left(\hat{h}\hat{D}_I\hat{\kappa}_a\hat{\Gamma}_m/(\hat{D}\hat{\kappa}_d)\right)^{1/2}$	m	$1.7 \cdot 10^{-4}$
Mobilization length (see Eq. 10)	$\hat{L}_m = (\hat{h}\hat{\kappa}_a\hat{\Gamma}_m/\hat{\kappa}_d)\left(n_s\hat{R}\hat{T}\hat{c}_0/(\hat{\mu}\hat{D})\right)^{1/2}$	m	$4.3 \cdot 10^{-2}$

Table SII. Dimensionless numbers governing the full problem.

Dimensionless group	Definition	Range in simulations	Value (or best estimate) in experiments
Normalized concentration	$k = \hat{\kappa}_a \hat{c}_0 / \hat{\kappa}_d = \hat{\Gamma}_0 / \hat{\Gamma}_m$	$2.7 \cdot 10^{-5} - 5.4 \cdot 10^{-2}$	$4 \cdot 10^{-2}$
Marangoni number	$Ma = n_s \hat{R}\hat{T}\hat{\Gamma}_m/(\hat{\mu}\hat{U})$	$3.1 \cdot 10^{3} - 2.3 \cdot 10^{7}$	$9 \cdot 10^{4}$
Péclet number	$Pe = \hat{h}\hat{U}/\hat{D}$	$1.5 \cdot 10^{-2} - 1.2 \cdot 10^{5}$	$2 \cdot 10^{1}$
Interface Péclet number	$Pe_I = \hat{h}\hat{U}/\hat{D}_I$	$1.7 \cdot 10^{-1} - 6 \cdot 10^{2}$	$2 \cdot 10^{1}$
Biot number	$Bi = \hat{h}\hat{\kappa}_d/\hat{U}$	$8.6 \cdot 10^{-3} - 2.5 \cdot 10^{2}$	$2 \cdot 10^{-1}$
Damköhler number	$Da = \hat{h}\hat{\kappa}_a\hat{\Gamma}_m/\hat{D}$	$2.5 \cdot 10^{1} - 6.4 \cdot 10^{3}$	$3 \cdot 10^{1}$
Normalized grating length	$g = \hat{g}/\hat{h} = \phi_x/\varepsilon$	$1.54 - 58.33$	$2.5 \cdot 10^{2} - 7.5 \cdot 10^{2}$
Normalized pitch	$P = \hat{P}/\hat{h}$	$0.92 - 2$	1
Streamwise gas fraction	ϕ_x	$0.833 - 0.994$	$0.9986 - 0.9995$
Spanwise gas fraction	ϕ_z	$0.667 - 0.980$	0.667
Normalized depletion length	$L_d = \hat{L}_d/\hat{h} = Da/(PeBi)$	$3.4 \cdot 10^{-3} - 2.7 \cdot 10^{4}$	7.8
Normalized modified depletion length	$L_d^{\text{mod}} = \hat{L}_d^{\text{mod}}/\hat{h} = (Da/(Pe_I Bi))^{1/2}$	$1.2 \cdot 10^{-1} - 4.9 \cdot 10^{1}$	2.8
Normalized mobilization length	$L_m = \hat{L}_m/\hat{h} = (k\, Ma\, Da/Bi)^{1/2}$	$2 - 1.1 \cdot 10^{3}$	$7 \cdot 10^{2}$

Velocity field. At leading order in the small parameter ε, Eqs. S3b-S3d representing the flow field are

$$\frac{\partial^2 u}{\partial y^2} + \frac{\partial^2 u}{\partial z^2} = \frac{\partial p}{\partial x}, \tag{S4a}$$

$$\frac{\partial p}{\partial y} = \frac{\partial p}{\partial z} = 0. \tag{S4b}$$

It is clear from [S4b] that p, and thus also $\partial_x p$, only depends on x. Since the solution u depends on x only through the right-hand-side of [S4a], we pose a piecewise solution

$$u(x,y,z) = \begin{cases} u_1(x,y,z) & \text{if } |x| < \phi_x/2, \\ u_2(x,y,z) & \text{if } \phi_x/2 \le |x| \le 1/2. \end{cases} \tag{S5}$$

Taking into account the boundary conditions, the function u_1 satisfies the mixed boundary-value problem given by [S4a]

and the boundary conditions

$$
\begin{aligned}
u_1 &= 0 \quad \text{if } y = 1 \text{ or if } y = -1 \text{ and } |z| \geq \phi_z P, \\
\frac{\partial u_1}{\partial y} &= \gamma(x) \quad \text{if } y = -1 \text{ and } |z| < \phi_z P.
\end{aligned}
\qquad \text{[S6]}
$$

We then introduce the Poiseuille profile $u_P(y) = (1 - y^2)/2$ and, by virtue of the linearity of the problem, decompose the solution following $u_1 = -\left[\partial_x p(x)\right] u_P(y) - \left[\gamma(x) + \partial_x p(x)\right] u_d^\infty$. The resulting problem for u_d^∞ is homogeneous, yielding

$$
\begin{aligned}
\frac{\partial^2 u_d^\infty}{\partial y^2} + \frac{\partial^2 u_d^\infty}{\partial z^2} &= 0, \\
u_d^\infty &= 0 \quad \text{if } y = 1 \text{ or if } y = -1 \text{ and } |z| \geq \phi_z P, \\
\frac{\partial u_d^\infty}{\partial y} &= -1 \quad \text{if } y = -1 \text{ and } |z| < \phi_z P.
\end{aligned}
\qquad \text{[S7]}
$$

The problem given by [S7] has been solved in closed form (8, 9), and highlights that $u_d^\infty(y, z)$ is simply the deviation from the Poiseuille profile in the *infinite-grating* problem.

The function u_2 satisfies [S4a] with the no-slip boundary conditions $u_2 = 0$ at $y = \pm 1$, and the solution is given by $u_2 = -\left[\partial_x p(x)\right] u_P(y)$. Consequently, the following linear combination of $u_P(y)$ and $u_d^\infty(y, z)$ solves [S4]:

$$
u(x, y, z) = \begin{cases} \left[-\dfrac{\partial p}{\partial x}(x)\right] u_P(y) - \left[\gamma(x) + \dfrac{\partial p}{\partial x}(x)\right] u_d^\infty(y, z) & \text{if } |x| < \phi_x/2, \\ \left[-\dfrac{\partial p}{\partial x}(x)\right] u_P(y) & \text{if } \phi_x/2 \leq |x| \leq 1/2. \end{cases}
\qquad \text{[S8]}
$$

To determine the pressure gradient term in [S8], we first pose a piecewise pressure field

$$
p(x) = \begin{cases} p_1(x) & \text{if } |x| < \phi_x/2, \\ p_2(x) & \text{if } \phi_x/2 \leq |x| \leq 1/2. \end{cases}
\qquad \text{[S9]}
$$

Integrating the continuity equation [S3a] across any cross section of the domain shows that the volumetric flow rate $Q = \int_{-P/2}^{P/2} \int_{-1}^{1} u(x, y, z)\, \mathrm{d}y\, \mathrm{d}z$ remains constant in x. Further integrating the piecewise solution [S8] and invoking [S9], we obtain two expressions for the flow rates

$$
\begin{aligned}
Q_1 &= \int_{-P/2}^{P/2} \int_{-1}^{1} u_1(x, y, z)\, dy\, dz = \left[-\frac{\partial p_1}{\partial x}(x)\right] \frac{2P}{3} - \left[\gamma(x) + \frac{\partial p_1}{\partial x}(x)\right] Q_d^\infty, \\
Q_2 &= \int_{-P/2}^{P/2} \int_{-1}^{1} u_2(x, y, z)\, dy\, dz = \left[-\frac{\partial p_2}{\partial x}\right] \frac{2P}{3},
\end{aligned}
$$

where $2P/3$ and $Q_d^\infty(\phi_z, P)$ are the flow rates given by $u_P(y)$ and $u_d^\infty(y, z)$, respectively. Since Q_2 must be constant in x, the pressure gradient $\partial_x p_2$ is necessarily independent of x as well. Equating $Q_1 = Q_2$ yields a relationship between the two pressure gradients,

$$
\frac{\partial p_1}{\partial x}(x) = \left[\frac{2P}{2P + 3Q_d^\infty}\right] \frac{\partial p_2}{\partial x} - \left[\frac{3Q_d^\infty}{2P + 3Q_d^\infty}\right] \gamma(x).
\qquad \text{[S11]}
$$

The last condition that must be satisfied by the solution is the fixed pressure drop across the domain given by [S1o]. The nondimensional version of this equation, taking $\alpha = 1$ and $\beta = 0$ in [S1o], leads to $p(x) = p(x + 1) + 1$. This equation can be made specific to $x = -1/2$ and recast into an integral equation for the gradient

$$
\int_{-1/2}^{1/2} \frac{\partial p}{\partial x}(x)\, \mathrm{d}x = -1
$$

which, after applying the decomposition [S9], leads to

$$
\int_{-1/2}^{-\phi_x/2} \frac{\partial p_2}{\partial x}\, \mathrm{d}x + \int_{-\phi_x/2}^{\phi_x/2} \frac{\partial p_1}{\partial x}(x)\, \mathrm{d}x + \int_{\phi_x/2}^{1/2} \frac{\partial p_2}{\partial x}\, \mathrm{d}x = -1.
\qquad \text{[S12]}
$$

Substituting [S11] into [S12], we arrive at

$$
\frac{\partial p_1}{\partial x}(x) = -\frac{2P + 3Q_d^\infty(1 - \phi_x)\langle\gamma\rangle}{2P + 3Q_d^\infty(1 - \phi_x)} + \frac{3Q_d^\infty}{2P + 3Q_d^\infty}(\langle\gamma\rangle - \gamma(x)), \qquad \frac{\partial p_2}{\partial x} = -\frac{2P + 3Q_d^\infty(1 - \phi_x\langle\gamma\rangle)}{2P + 3Q_d^\infty(1 - \phi_x)},
\qquad \text{[S13]}
$$

which, after defining $q_d^\infty = 3Q_d^\infty/(2P)$, can finally be substituted into [S8] to produce the closed form solution for the flow field Eq. 1 in the main text. The term $\langle\gamma\rangle$ in [S13] represents the average value of $\gamma(x)$ at the interface, i.e. $\langle\gamma\rangle = \frac{1}{\phi_x} \int_{-\phi_x/2}^{\phi_x/2} \gamma(x)\, \mathrm{d}x$.

Once the leading-order velocity field [S8] is fully determined from known parameters, the relevant quantities characterizing the performance of the SHS can be readily obtained. The *local* centerline slip velocity $u_{Ic} = u(x, y = -1, z = 0)$ is

$$u_{Ic}(x) = 2P\left[\frac{(1-\langle\gamma\rangle)}{2P + 3Q_d^\infty(1-\phi_x)} + \frac{\langle\gamma\rangle - \gamma(x)}{2P + 3Q_d^\infty}\right] u_{Ic}^\infty, \qquad \text{[S14]}$$

with $u_{Ic}^\infty(\phi_z, P) = u_d^\infty(y = -1, z = 0)$. With the additional assumption of a uniform shear stress $\gamma(x) = \langle\gamma\rangle$, justified in Section *Scaling theory for surfactant transport*, [S14] further simplifies to

$$u_{Ic} = \left[\frac{2Pu_{Ic}^\infty}{2P + 3Q_d^\infty(1-\phi_x)}\right](1-\langle\gamma\rangle) := u_{Ic}^{\text{clean}}(1-\langle\gamma\rangle), \qquad \text{[S15]}$$

where we define $u_{Ic}^{\text{clean}}(\phi_x, \phi_z, P)$ as the centerline slip velocity for the finite-grating *clean* case (i.e. $\gamma(x) = \langle\gamma\rangle = 0$). We show in Section *Scaling theory for surfactant transport* that we can model the average shear stress $\langle\gamma\rangle$ using a scaling analysis of the surfactant transport equations, leading to Eq. S28. Introducing [S28] into [S15], we arrive at Eq. 3 of the main text. Another common, *global* measure of SHS performance is the increase in flow rate with respect to that of a Poiseuille flow. Our theory predicts

$$Q_d = \int_{-P/2}^{P/2}\int_{-1}^{1}[u(x,y,z) - u_P(y)]\,\mathrm{d}y\,\mathrm{d}z = \left[\frac{2P\phi_x Q_d^\infty}{2P + 3Q_d^\infty(1-\phi_x)}\right](1-\langle\gamma\rangle) := Q_d^{\text{clean}}(1-\langle\gamma\rangle), \qquad \text{[S16]}$$

where we again introduce $Q_d^{\text{clean}}(\phi_x, \phi_z, P)$ as the increase in flow rate for the finite-grating, clean problem. Perhaps the most common global quantity sought in theoretical SHS studies is the effective slip length λ_e. Here λ_e is defined as the quantity that yields the same increase Q_d in flow rate if the mixed boundary conditions on $y = -1$ are replaced with a uniform Navier-slip condition $u = \lambda_e \partial_y u$. Such a flow yields a solution $u_{\lambda_e}(y) = u_P(y) + \lambda_e(1-y)/(2+\lambda_e)$ and thus an increase in flow rate of $2P\lambda_e/(2+\lambda_e)$ which, when equated to Q_d, yields an expression for the slip length

$$\lambda_e = \frac{2Q_d}{2P - Q_d} = \frac{2\phi_x Q_d^\infty(1-\langle\gamma\rangle)}{2P + [3 - \phi_x(4-\langle\gamma\rangle)]\,Q_d^\infty}. \qquad \text{[S17]}$$

Infinite-grating problem. The calculation of Q_d^∞ and u_{Ic}^∞ for any values of P and ϕ_z require either the numerical solution of a dual trigonometric series (9) or the solution of nonlinear algebraic equations involving elliptic integrals and elliptic functions (8). We will not provide such level of detail here and instead refer the reader to (8–10). However, for completeness we provide the asymptotic limits

$$Q_d^\infty \to \frac{P^2}{\pi}\ln\left(\sec\left(\frac{\pi\phi_z}{2}\right)\right) \quad \text{for } P \ll 1, \qquad \text{[S18a]}$$

$$Q_d^\infty \to 2\phi_z P \quad \text{for } P \gg 1, \qquad \text{[S18b]}$$

and

$$u_{Ic}^\infty \to \frac{P}{\pi}\operatorname{arccosh}\left(\sec\left(\frac{\pi\phi_z}{2}\right)\right) \quad \text{for } P \ll 1, \qquad \text{[S19a]}$$

$$u_{Ic}^\infty \to 2 \quad \text{for } P \gg 1. \qquad \text{[S19b]}$$

Since the values of Q_d^∞ and u_{Ic}^∞ are monotonically increasing with P, the above limit [S19] reveals that $u_{Ic}^\infty < 2$. In addition, the limits [S18], [S19] can be combined to obtain useful approximations for all P, which are within 12% of the exact values if $0.5 < \phi_z < 0.95$, and are of course most accurate at large or small P:

$$Q_d^\infty \approx \left\{\left[\frac{P^2}{\pi}\ln\left(\sec\left(\frac{\pi\phi_z}{2}\right)\right)\right]^{n_q} + [2\phi_z P]^{n_q}\right\}^{1/n_q}, \quad n_q \approx -1.46 \qquad \text{[S20a]}$$

$$u_{Ic}^\infty \approx \left\{\left[\frac{P}{\pi}\operatorname{arccosh}\left(\sec\left(\frac{\pi\phi_z}{2}\right)\right)\right]^{n_u} + 2^{n_u}\right\}^{1/n_u}, \quad n_u \approx -1.21. \qquad \text{[S20b]}$$

Scaling theory for surfactant transport

Full problem. The analysis of the surfactant transport equations is similar to that in (11), which we recapitulate here in order to clarify the differences between the two-dimensional and three-dimensional cases. The first assumption of our model for [S3e]-[S3k] is that the concentration of surfactant is low enough to ensure a *dilute regime*, that is $k \ll 1$. We expect this assumption to be the case for most situations in which surfactants are not artificially added, for instance, when unwanted contaminants are naturally present in water (as discussed in (11)). Additionally, since the interaction parameter A is typically not large in absolute value, with $|A| \lesssim 20$ (2), it is possible to assume that $k|A| \ll 1$ as well. The nonlinear terms [S3j] and [S3k] in the governing equations can then be linearized, leading to Henry kinetics, that is $\mathcal{S}(c_I, \Gamma) = c_I - \Gamma + O(k) + O(k|A|)$

and $\mathcal{N}(\Gamma) = 1 + O(k) + O(k|A|)$. Consequently, at leading order in k and $k|A|$, [S3f]–[S3k] can be simplified, yielding Eq. 2 in the main text.

Applying an integral average to [S3f] along the spanwise direction, we obtain

$$\frac{\partial \langle u_I \Gamma \rangle_z}{\partial x} = \frac{\varepsilon}{Pe} \frac{\partial^2 \langle \Gamma \rangle_z}{\partial x^2} + \frac{Bi}{\varepsilon} (\langle c_I \rangle_z - \langle \Gamma \rangle_z), \tag{S21}$$

where the spanwise average across the plastron of a function $f(z)$ is defined as $\langle f \rangle_z = \frac{1}{\phi_z P} \int_{-\phi_z P/2}^{\phi_z P/2} f(z)\,\mathrm{d}z$, and where the terms in [S3f] associated with derivatives in z vanish due to the no-slip ($w = 0$) and no-flux ($\partial_z \Gamma = 0$) boundary conditions at the edges $z = \pm\phi_z P/2$ of the plastron. If [S21] is further integrated from $x = -\phi_x/2$ to $x = \phi_x/2$ and boundary conditions $u = 0$ and $\partial_x \Gamma = 0$ are applied at $x = \pm\phi_x/2$, we have that

$$\int_{-\phi_x/2}^{\phi_x/2} (\langle c_I \rangle_z - \langle \Gamma \rangle_z)\,\mathrm{d}z = 0, \tag{S22}$$

and thus, by virtue of the mean value theorem, an equilibrium condition $\langle c_I \rangle_z = \langle \Gamma \rangle_z$ must occur at some coordinate along the interface, which we call x_0. Downstream from x_0, the flow advection promotes the accumulation of interfacial surfactant, which in turn triggers a net desorption flux and an increase in bulk surfactant with respect to the background level. Upstream from x_0, the situation is the opposite, with a deficit of Γ and c_I with respect to the equilibrium values and a net adsorption flux. Figure 1C, D depicts this physical scenario with the two distinct regions along the interface.

The second main assumption is to consider the interfacial concentration Γ as approximately linear. In this case, [S22] implies that the equilibrium point must be approximately at the center of the interface (i.e. $x_0 \approx 0$), and thus the bulk concentration at x_0 is approximately the background concentration and we have $\langle c \rangle_z(x_0) = \langle \Gamma \rangle_z(x_0) \approx 1$. Consequently, this assumption allows to scale the concentrations at both ends of the interface $x = \pm\phi_x/2$ as

$$c(x = \pm\phi_x/2) \sim 1 \pm \Delta c, \tag{S23a}$$

$$\Gamma(x = \pm\phi_x/2) \sim 1 \pm \Delta\Gamma, \tag{S23b}$$

with Δc and $\Delta\Gamma$ the characteristic variation of the concentrations (see Fig. 1C, D). Additionally, note that an approximately linear Γ also implies, from [S3h], that the Marangoni shear at the interface is approximately constant (i.e. $\gamma(x) \approx \langle \gamma \rangle$). This assumption is expected to hold as long as the flow is not in the so-called stagnant cap regime (11), characterized by a strongly nonuniform interfacial concentration. The stagnant cap regime is reached when advection at the interface overcomes both diffusion and kinetic effects (12), that is, when $\varepsilon Pe_I \gg 1$ and either $Bi/\varepsilon \ll 1$ or $Da \gg 1$ (11). Given the typical parameter values in small-scale flows like the ones considered in this study (see Section *Estimate of surfactant parameters* and Table SII), we conclude that for long gratings we have $\varepsilon Pe_I \lesssim 1$, which justifies the assumption of an approximately linear Γ. Furthermore, we verify a posteriori that our simulation results show an approximately linear profile of Γ (see Section *Finite-element simulations*).

Using these two assumptions, it is possible to use scaling arguments on [S3] to obtain an expression for $\langle \gamma \rangle$ as a function of the nondimensional groups of the problem. We start by scaling the terms in [S3h] as $\partial_y u|_I \sim \langle \gamma \rangle$ and $\partial_x \Gamma \sim \Delta\Gamma/\phi_x$, leading to

$$\Delta\Gamma \sim \frac{\phi_x \langle \gamma \rangle}{\varepsilon k Ma}. \tag{S24}$$

Next, we evaluate the terms in [S3j] at the interface ends $x = \pm\phi_x/2$. We take $\partial_y c|_I \sim [1 - (1 \pm \Delta c_I)]/\delta \sim \mp\Delta c_I/\delta$ and $(c_I - \Gamma) \sim [1 \pm \Delta c_I - (1 \pm \Delta\Gamma)] \sim \pm(\Delta c_I - \Delta\Gamma)$, where $\delta = \hat{\delta}/\hat{h}$ is the characteristic boundary layer thickness of the bulk concentration (Fig. 1C). We arrive at

$$\Delta c_I \sim \frac{\delta Da}{(1 + \delta Da)} \frac{\phi_x \langle \gamma \rangle}{\varepsilon k Ma}. \tag{S25}$$

Eq. S21 is integrated from $x = -\phi_x/2$ to $x = x_0$, leading to

$$\langle u_I \Gamma \rangle_z(x_0) = \frac{\varepsilon}{Pe_I} \frac{\partial \langle \Gamma \rangle_z}{\partial x}(x_0) + \frac{Bi}{\varepsilon} \int_{-\phi_x/2}^{x_0} (\langle c_I \rangle_z - \langle \Gamma \rangle_z)\,\mathrm{d}x, \tag{S26}$$

whose terms we scale as $\langle u_I \Gamma \rangle_z(x_0) \sim u_{Ic}$, $\partial_x \langle \Gamma \rangle_z(x_0) \sim \Delta\Gamma/\phi_x$, and $\int_{-\phi_x/2}^{x_0} (\langle c_I \rangle_z - \langle \Gamma \rangle_z)\,\mathrm{d}x \sim \phi_x(\Delta c_I - \Delta\Gamma)$. Using [S24] and [S25] and introducing $g = \hat{g}/\hat{h} = \phi_x/\varepsilon$, we arrive at

$$u_{Ic} \sim \frac{\langle \gamma \rangle}{kMa} \left(\frac{1}{Pe_I} + \frac{Bi\, g^2}{(1 + \delta Da)} \right),$$

which, after introducing empirical coefficients for each term, yields

$$u_{Ic} = \frac{\langle \gamma \rangle}{a_1 kMa} \left(\frac{1}{Pe_I} + a_2 \frac{Bi\, g^2}{(1 + \delta Da)} \right). \tag{S27}$$

Making use of the theory for the flow field [S15], we substitute $u_{Ic} = (1 - \langle\gamma\rangle) u_{Ic}^{\text{clean}}$ into [S27] and obtain the expression for $\langle\gamma\rangle$ as a function of the parameters of the problem,

$$\langle\gamma\rangle = \frac{a_1\, k\, Ma\, u_{Ic}^{\text{clean}}}{\dfrac{1}{Pe_I} + a_1\, k\, Ma\, u_{Ic}^{\text{clean}} + a_2 \dfrac{Bi\, g^2}{(1+\delta Da)}}. \quad \text{[S28]}$$

Equation S28 can now be introduced in [S14] to obtain the formula for the slip velocity [3] in the main text. Similarly, combining [S28] with [S16] and [S17], expressions for the increase in flow rate and effective slip length can be reached.

The only yet undetermined part of the model is an expression for the boundary layer thickness δ, which we seek through scaling of the conservation law for the bulk surfactant [S3e]. In situations with $\varepsilon Pe \gg 1$, streamwise advection must balance wall-normal diffusion $u\partial_x c \sim \frac{1}{\varepsilon Pe}\partial_{yy} c$, which is only possible if c varies over a small length scale $\delta \ll 1$ (12). We take $\partial_x c \sim \Delta c_I/\phi_x$, $\partial_{yy} c \sim \Delta c_I/\delta^2$ and the velocity inside the boundary layer as $u \sim u_{Ic} + \langle\gamma\rangle\delta$. In the case of an interface close to immobilization, i.e. $u_{Ic} \sim 0$ and $\langle\gamma\rangle \sim 1$, these scalings indicate that $\delta \sim (Pe/g)^{-1/3}$ when $\varepsilon Pe \gg 1$. In the opposite case of $\varepsilon Pe \ll 1$, Eq. S3e is dominated by diffusion, and thus the characteristic length scale of variation of c in the wall-normal direction is the whole half height of the domain, implying $\delta \sim 1$. We choose

$$\delta = a_3 (1 + a_4 Pe/g)^{-1/3} \quad \text{[S29]}$$

to satisfy these two extremes, with a_3 and a_4 empirical parameters. It is also possible to obtain a similar expression with an exponent of $-1/2$ instead, by assuming that the boundary layer is essentially shear-free (i.e. $u_{Ic} \sim 1$ and $\langle\gamma\rangle \sim 0$). In practice, the overall value of quantities like u_{Ic} are only weakly dependent on the specific functional form of δ, so we only consider the expression [S29]. Additionally, in the case of interest of long gratings $\varepsilon \ll 1$ in small-scale flows we typically have $Pe/g \lesssim 1$ (Section *Estimate of surfactant parameters*) and thus the boundary layer thickness is approximately independent of Pe or g.

Insoluble surfactant limit. All previous theoretical expressions can also be obtained in the case of an insoluble surfactant, i.e. taking $\mathcal{S}(c_I, \Gamma) = 0$ in [S3g] and neglecting [S3e] and [S3j]. In this case, $\hat{\Gamma}_0$ is an independent parameter that is not linked to $\hat{c}_0$, since the bulk concentration is undefined for an insoluble surfactant. The value of k is now simply $k = \hat{\Gamma}_0/\hat{\Gamma}_m$, although we assume $k \ll 1$ still holds and leads to $\mathcal{N}(\Gamma) \approx 1$. Furthermore, since $\varepsilon Pe_I \lesssim 1$ remains valid we can still assume a regime away from the stagnant cap and thus an approximately linear profile for Γ. The same steps taken for the scaling of [S3h] and [S21] can be followed to arrive at

$$\langle\gamma\rangle = \frac{a_{\text{ins}}\, Ma_{\text{ins}}\, u_{Ic}^{\text{clean}}}{1 + a_{\text{ins}}\, Ma_{\text{ins}}\, u_{Ic}^{\text{clean}}},$$

$$u_{Ic} = \frac{u_{Ic}^{\text{clean}}}{1 + a_{\text{ins}}\, Ma_{\text{ins}}\, u_{Ic}^{\text{clean}}},$$

$$\lambda_e = \frac{\lambda_e^{\text{clean}}}{1 + a_{\text{ins}}\, Ma_{\text{ins}}\, u_{Ic}^{\text{clean}} \left(1 + \frac{\lambda_e^{\text{clean}}}{2}\right)},$$

with $Ma_{\text{ins}} = k\, Ma\, Pe_I = n_s \hat{R}\hat{T}\hat{\Gamma}_0 \hat{h}/(\hat{\mu}\hat{D}_I)$, and a_{ins} another empirical parameter.

Finite-element simulations

We solve numerically the full governing equations [S1a]-[S1t] of the problem in dimensional form, performing a total of 155 simulations. The objectives are to (i) determine the values of the empirical parameters a_1, a_2, a_3 and a_4 in our model (Section *Scaling theory for surfactant transport*), (ii) confirm the modeling assumptions, for the interfacial concentration Γ, of an approximately constant profile in the spanwise direction, and of a linear profile in the streamwise direction (Section *Scaling theory for surfactant transport*), and (iii) compare the theory to simulations of realistic microchannels in conditions representative of our experiments (Section *Experimental methods*).

We implemented the three-dimensional simulations using the finite-element software COMSOL Multiphysics 5.5®. The simulation domain is one half of the SHS unit cell depicted in Fig. 1, with $\hat{z}$ spanning only between $\hat{z} = 0$ and $\hat{z} = \hat{P}/2$ due to the spanwise symmetry of the solution. The volume is meshed with tetrahedral elements, concentrating the finest regions around the upstream and downstream edges of the interface $\hat{x} = \pm\phi_x \hat{L}/2$ since it is in those areas where the most abrupt variations of the solution occur (see Fig. S1). Across all the simulations, the minimum element size (understood as the diameter of a sphere circumscribing the smallest element) is set to $1.5 \cdot 10^{-9}$ m.

The solution of the governing equations is achieved with a combination of the Creeping Flow module for the flow equations [S1a]-[S1d] and the Dilute Species Transport module for the transport of bulk surfactant [S1e]. The conservation law for the interfacial surfactant [S1f] is implemented through a General Form Boundary PDE, using [S1j] as the source term. The Marangoni boundary conditions [S1h] and [S3i] are enforced through a Weak Contribution constraint, as is the condition that fixes the mean bulk concentration [S1l].

The system of nonlinear equations is solved through a Newton-type iterative method using the PARDISO direct solver for the linear system at each iteration. We set a relative tolerance of 10^{-5} as a convergence criterion for the solution, which is

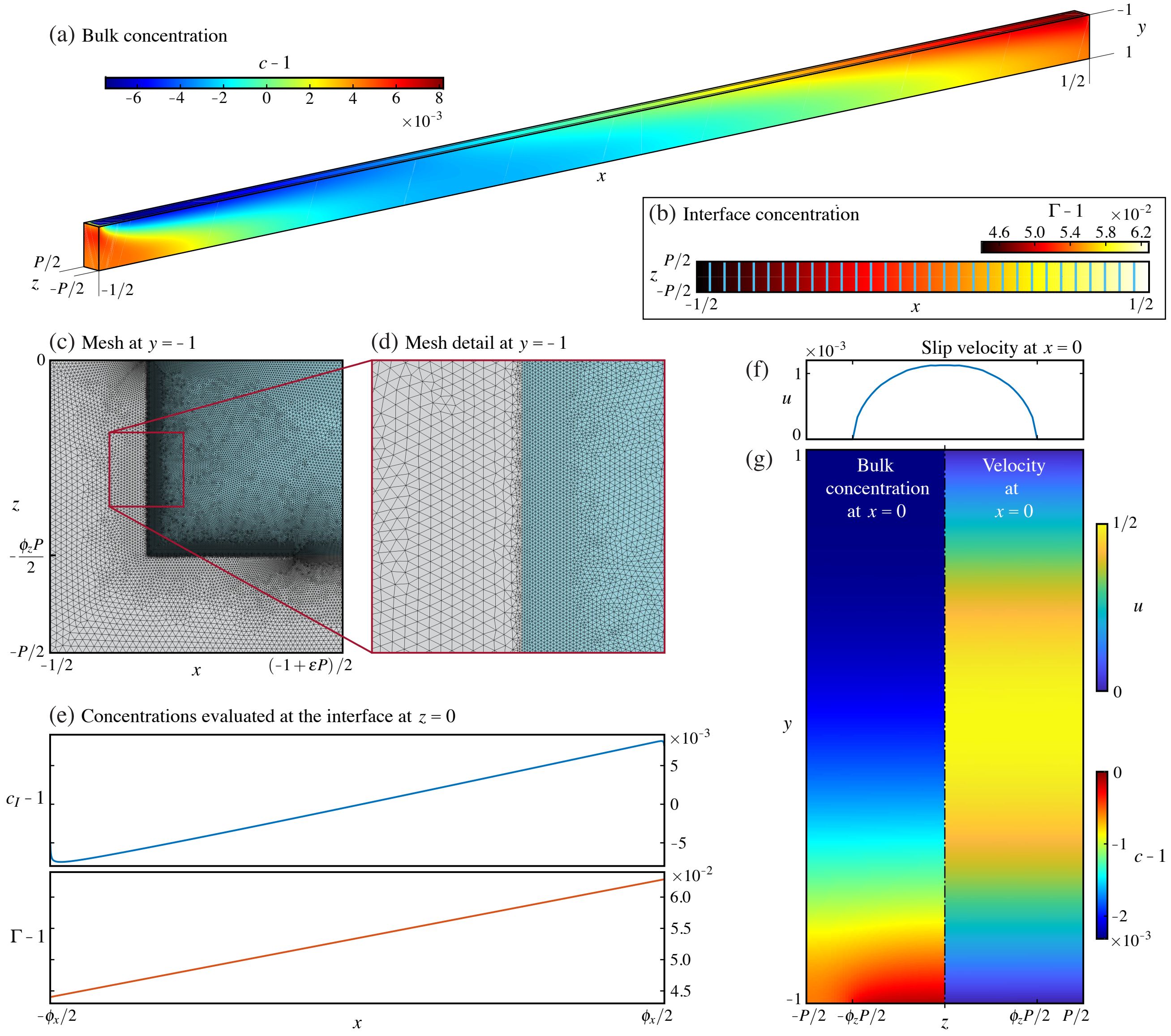

Fig. S1. Results from the finite-element numerical simulations, obtained with the parameter values that were estimated for the experiments (third column of Table SI), using a grating length of $\hat{g} = 3.5\,\mathrm{mm}$ due to limitations in computing power. Note that the assumption of a spanwise constant interface concentration is satisfied as shown in (b). In addition, the profiles of c_I and Γ remain approximately linear as shown in (e). Despite the nonzero slip velocity shown in (f), the velocity profile is very close to a purely parabolic Poiseuille flow in (g), since at this grating length the interface is nearly immobilized.

satisfied by all of our simulations. The pressure, bulk concentration and interfacial concentration are discretized using linear elements, and the velocity field uses either quadratic or linear elements, depending on the computational demands of each simulation.

We vary the problem parameters to ensure that each of the distinct terms that are pre-multiplied by an empirical coefficient in [S28] changes over a few orders of magnitude. The ranges of variation of each dimensional quantity in the simulations, as well as of the corresponding nondimensional numbers, is indicated in Tables SI and SII. A small number of simulations were chosen with the same parameters as those estimated in the experiments, in order to achieve a direct comparison (see Fig. 3 in the main text). However, due to constraints in computational power, the value of the grating length $\hat{g}$ was smaller than that of the microfluidic devices.

The parameters a_1, a_2, a_3 and a_4 are obtained through least-squares fitting using the MATLAB function `lsqnonlin`. We define the error as the total sum of squares of the difference between the centerline slip velocities computed in the simulations and those predicted by the theory [3], i.e. $\mathrm{ERR} = \sum (u_{Ic}^{\mathrm{theory}} - u_{Ic}^{\mathrm{sim}})^2$. We find $a_1 = 0.345$, $a_2 = 0.275$, $a_3 = 5.581$, and $a_4 = 3.922$. As illustrated in Fig. 2A, the agreement between simulations and theory is excellent over more than four orders of magnitude in the slip velocity.

Experimental methods

The experimental setup is centered around the custom-built microfluidic device depicted in Fig. 3A–C. The chips are made by casting PDMS (Sylgard 184) with a 1:10 mass ratio of elastomer-to-curing agent, using a master mold fabricated by two-layer photolithography. The photoresist used for the mold is SU-8 (Microchem SU-8 3050 for the first layer, corresponding to the main channel, and Microchem SU-3025 for the second layer, corresponding to the gratings). The flat PDMS substrate is determined to be hydrophobic, with static contact angles of water droplets measured to be higher than 90°. The textured PDMS exhibits a further increase of the static contact angle, with air pockets within the texture that are observable under the microscope, demonstrating that the PDMS textured substrate is in a Cassie-Baxter superhydrophobic state. Measured values of static contact angles are consistent with previous measurements for untreated PDMS (13).

The chip is bonded to a 0.1 mm-thick glass coverslip (Bellco Glass 1916-25075) through untreated adhesion, and a 40X water objective is used to image the interior of the channels through the coverslip using a confocal microscope (Leica SP8 Resonant Scanning). The device is placed inside a stage top chamber (Okolab H101-K-FRAME) that ensures precise control of temperature, which we set to $\hat{T} = 296\,\mathrm{K}$. The fluid is initially contained in a glass syringe (Hamilton Gastight), and driven by a syringe pump (KD Legato 111) at a constant flow rate through plastic tubing (Tygon S3) into and out of the microfluidic channel. We use the barrel of a plastic syringe (BD Luer-Lok) as an outlet reservoir open to the room. To ensure that the air-water interface in the observed channels remains flat and that plastron curvature effects can be safely neglected, the magnitude of the pressure inside the channel is adjusted by varying the height of the outlet reservoir, which is mounted on a vertical translation stage (Thorlabs VAP10). The maximum deflection of the interface at the centerline, relative to the edges, is estimated to be less than $\pm 1\,\mu\mathrm{m}$.

Due to the extreme difficulty of removing all traces of surface-active contaminants, even in controlled experimental conditions (4), we do not attempt an exhaustive cleaning protocol with that aim. Nevertheless, we follow standard cleaning procedures on all syringes and tubing, ensuring that they are rinsed with $18\,\mathrm{M\Omega\,cm}$ DI water with at least twice their volume before they are used. In addition, we follow a specific cleaning protocol for the μ-PIV particles (ThermoFisher FluoSpheres carboxylate 0.5-µm diameter), since they typically contain surfactants to prevent particle aggregation (14). We use a centrifuge (Eppendorf 5418) to separate the beads from the buffer solution, which is discarded and replenished with clean $18\,\mathrm{M\Omega\,cm}$ DI water, and we repeat the process three times. These cleaning procedures ensure that the traces of surfactants responsible for the non-negligible Marangoni stresses that we observe in the experiments are the result of contamination that would naturally occur in typical small-scale flows through microfluidic devices, and not as a byproduct of the specific experimental methods used in this study.

The μ-PIV analysis is performed using the open-source MATLAB toolbox `PIVlab`. The acquisition window has an approximate size of $125\,\mu\mathrm{m} \times 125\,\mu\mathrm{m}$, which is sufficient to cover the span of two pitches (see Fig. 3A,B,C), which are imaged around the center of the grating in the streamwise direction (i.e. $x = 0$). We image the motion of the μ-PIV particles across time intervals between 20 s and 60 s, at different distances from the interface, with frame rates between 20 fps and 28 fps.The velocity field is averaged in time and in the streamwise (x) direction to obtain the velocity profiles depicted in Fig. 3D. To calculate the value of $\hat{u}_{Ic}$, we perform a linear least-squares fit, typically using between three and five velocity profiles to obtain an extrapolated slip velocity, from which we extract its value at $z = 0$. This linear fit is performed in MATLAB with a script that takes into account the propagation of uncertainties in the distance $\Delta\hat{y}$ from the interface, as well as the uncertainty in $\hat{u}$ inherent to the measurement and the averaging in the x direction.

Estimate of surfactant parameters

The main challenge in comparing the experimental measurements of $\hat{u}_{Ic}$ to the predictions from our model is the absence of information regarding the type and amount of surfactant present in the channels. Some parameters in the problem are known from the experimental conditions, and hence we fix those as $n_s = 2$ (3), $\hat{R} = 8.314\,\mathrm{J\,mol^{-1}\,K^{-1}}$, $\hat{T} = 296\,\mathrm{K}$ and $\hat{\mu} = 8.9 \cdot 10^{-4}\,\mathrm{kg\,m^{-1}\,s^{-1}}$ (15). Others can be accurately estimated, since most surfactants have diffusivities (both $\hat{D}$ and $\hat{D}_I$) bounded between $5 \cdot 10^{-10}$ and $5 \cdot 10^{-9}\,\mathrm{m^2\,s^{-1}}$, and have values of $\hat{\Gamma}_m$ between $1 \cdot 10^{-6}$ and $5 \cdot 10^{-5}\,\mathrm{mol\,m^{-2}}$ (2). We thus use as a reference surfactant the well-studied sodium dodecyl sulfate (SDS), setting $\hat{D} = \hat{D}_I = 7 \cdot 10^{-10}\,\mathrm{m^2\,s^{-1}}$ and $\hat{\Gamma}_m = 3.9 \cdot 10^{-6}\,\mathrm{mol\,m^{-2}}$. However, the values of the rate constants $\hat{\kappa}_a$ and $\hat{\kappa}_d$ can vary significantly across surfactants (2), and they cannot be assumed a priori. In addition, we must estimate the background bulk concentration $\hat{c}_0$.

Here we describe a strategy to estimate these quantities. The value of $k = \hat{\kappa}_a\hat{c}_0/\hat{\kappa}_a$ has an upper bound, since we expect $k \ll 1$ not only because this is the case when surfactants are not artificially added, but also because values of $k \gtrsim 1$ would significantly decrease the mean surface tension, leading to a rapid plastron collapse, which is not observed in experiments. We therefore choose the bound $k < k_{\max} = 1 \cdot 10^{-1}$, which ensures that k remains at least one order of magnitude smaller than 1 and that the absolute surface tension decrease $\Delta\hat{\sigma}$ is small compared to the clean surface tension value $\hat{\sigma}_0 = 7.2 \cdot 10^{-2}\,\mathrm{N\,m^{-1}}$. Indeed, an estimation using an equation of state derived from the Langmuir isotherm (11) yields $\Delta\hat{\sigma}/\hat{\sigma}_0 = n_s\hat{R}\hat{T}\hat{\Gamma}_m \ln(1 + k_{\max})/\hat{\sigma}_0 \approx 0.025$.

To establish a lower bound for k, we note that a near-immobilized plastron, as in the experiments in (4), requires that $\varepsilon k Ma \Delta\Gamma/\phi_x = k Ma \Delta\Gamma/g \approx 1$, from a scaling analysis of [S3h]. In conditions far away from the stagnant cap regime, the increase of interfacial surfactant $\Delta\Gamma$ can also be bounded $\Delta\Gamma < 1$, giving $k Ma/g \gtrsim 1$. This leads to a lower bound for k given by $k > k_{\min} = g/Ma$ which, using the values $g = 600$ and $Ma \approx 8.3 \cdot 10^4$ (estimated using $\hat{\Gamma}_m = 3.9 \cdot 10^{-6}\,\mathrm{mol\,m^{-2}}$ and $\hat{U} \approx 3\langle\hat{u}\rangle_{yz} = 2.61 \cdot 10^{-4}\,\mathrm{m\,s^{-1}}$ from (4), where $\langle\hat{u}\rangle_{yz}$ is the velocity average across the yz-plane), yields

$$7.3 \cdot 10^{-3} \lesssim k \lesssim 1 \cdot 10^{-1}. \qquad \text{[S31]}$$

In addition, the expression [S27] can be combined with the quantitative results from (4) to estimate the kinetic rate constants $\hat{\kappa}_a$ and $\hat{\kappa}_d$. For long gratings (i.e. $g \to \infty$), [S27] indicates that the slip velocity converges to the clean-case value $u_{Ic} \to u_{Ic}^{\text{clean}}$ (see Fig. 3F). However, for intermediate lengths in which u_{Ic} is still close to u_{Ic}^{clean}, the dominant balance of terms in [S27] yields the approximation $u_{Ic} \approx a_2 Bi\, g^2/[a_1 k Ma(1+\delta Da)]$. Moreover, the estimated order of magnitude of $\hat{D}_I$ results in a thick boundary layer $\delta \approx a_3$, since $Pe/g = O(10^{-2}) \ll 1$ when the values $\hat{h} = 5 \cdot 10^{-5}$ m, $\hat{U} \approx 3\langle \hat{u} \rangle_{yz} = 2.61 \cdot 10^{-4}$ m s^{-1} and $g = 600$ from (4) are used. The value of $\hat{\kappa}_a \hat{\Gamma}_m$ is generally large enough to guarantee $\hat{\kappa}_a \hat{\Gamma}_m > 10^{-5}$ m s^{-1} (2), which suggests that $Da \gg 1$. This means that $(1 + Da\,\delta) \approx a_3 Da$, yielding

$$k \frac{\hat{\kappa}_a}{\hat{\kappa}_d} \approx \frac{a_2 g^2}{a_1 a_3 Ma} \left(\frac{\hat{D}}{\hat{u}_{Ic} \hat{\Gamma}_m} \right). \qquad \text{[S32]}$$

We use $\hat{u}_{Ic} = 12.18 \pm 3.48\,\mu\text{m s}^{-1}$ from (4) in the right-hand side of [S32]; the other parameters are already known or estimated. Combining this expression with the bounds [S31] for k, we obtain

$$7.1 \cdot 10^1 \,\frac{\text{m}^3}{\text{mol}} \lesssim \frac{\hat{\kappa}_a}{\hat{\kappa}_d} \lesssim 1.8 \cdot 10^3 \,\frac{\text{m}^3}{\text{mol}}. \qquad \text{[S33]}$$

As mentioned previously, the Damköhler number is high for most surfactants (2), so the values of $\hat{\kappa}_a$ and $\hat{\kappa}_d$ have a weak effect individually, and the ratio $\hat{\kappa}_a/\hat{\kappa}_d$ from [S33] is enough to characterize the surfactant. The grey band in Fig. 3F corresponds to the bounds set by [S31] and [S33] in the limit of $Da \to \infty$. The edges of the band change only slightly when values of Da as low as 1 are considered.

The specific choice of $\hat{c}_0 = 3 \cdot 10^{-4}$ mol m^{-3}, $\hat{\kappa}_a = 89.5$ m^3 mol^{-1} s^{-1} and $\hat{\kappa}_d = 0.75$ s^{-1} (which leads to $k = 3.58 \cdot 10^{-2}$ and $\hat{\kappa}_a/\hat{\kappa}_d = 1.19 \cdot 10^2$ m^3 mol^{-1}) yields a good agreement with our experimental results (Fig. 3F).

Discussion of the mobilization length

Figures S2A, B display the same data collapse as Figs. 4B, C, but include the data from all 155 simulations. The yellow triangles indicate simulations with a thin boundary layer $\delta < 1$, which can only occur if $Pe/g \gg 1$. In practical applications with long gratings $g \gg 1$, such a regime would require extremely large Péclet numbers $Pe \gg g \gg 1$ that are outside the scope of this study. The green diamonds denote cases in which the grating length is below the modified depletion length $g < L_d^{\text{mod}}$, which are of little practical relevance. This is due to the fact that, for typical surfactants, we find $L_m \gg L_d^{\text{mod}}$ (see Section *Problem parameters*), and thus L_m is ultimately the lengthscale that g needs to overcome for a SHS grating to display significant slip.

Figure S2C shows the same data collapse when the parameter $(u_{Ic}^{\text{clean}})^{1/2}$ is omitted from the normalization of g. Note that u_{Ic}^{clean} depends only on the SHS texture geometry. Since, for SHS capable of drag reduction, one needs u_{Ic}^{clean} of order one (see Section *Infinite-grating problem*), the collapse in Fig. S2C is only marginally worse than in Fig. S2B. Furthermore, a good approximation to the curve shown in Fig. S2B is found by using Eq. 11 with $u_{Ic}^{\text{clean}} = 1/2$, as shown in Fig. S2C.

For convenience, here we provide a detailed discussion of the assumptions under which the surfactant problem is only a function of g/L_m:

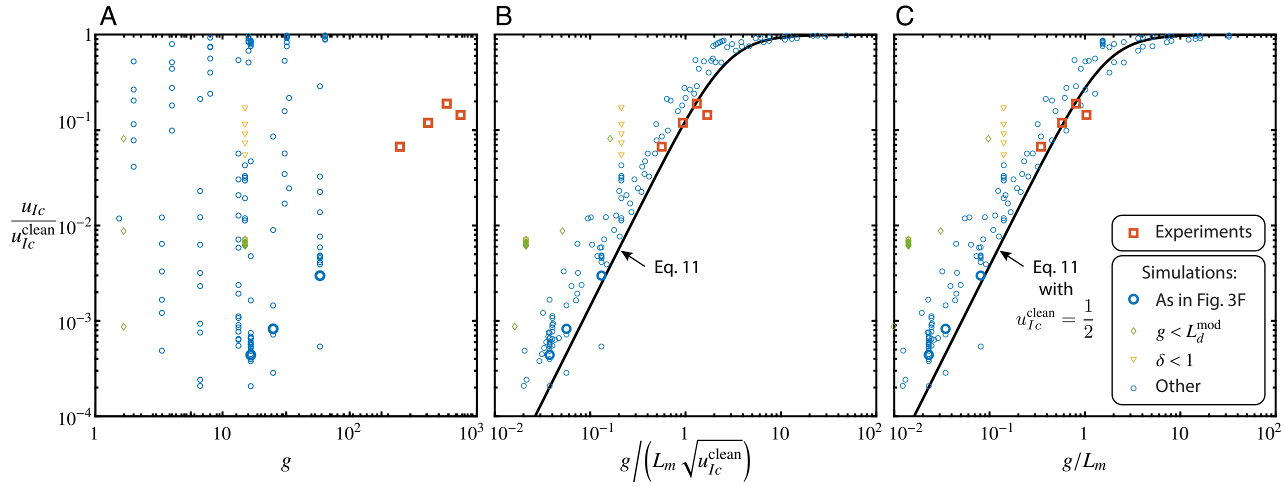

Fig. S2. (A) and (B): Collapse of slip velocity data after normalization with the mobilization length (as in Figs. 4B, C), including all 155 simulations. Yellow triangles are simulations with a thin boundary layer $\delta < 1$, which is not representative of long gratings, whereas the green diamonds denote cases where the grating length is below the modified depletion length $g < L_d^{\text{mod}}$, which are of little practical relevance, as explained in the text. (C) Data collapse when u_{Ic}^{clean} is ignored in the normalization of g. The curve shows Eq. 11 with a constant value of $u_{Ic}^{\text{clean}} = 1/2$.

- Dilute surfactant regime, $k \ll 1$: This is consistent with environmental traces of surfactant, intrinsic to the microfluidic system, without artificially added surfactant. Furthermore, for values of $k \sim O(1)$ the plastron would risk collapse due the decrease in mean surface tension. Based on estimates from experimental data (4), we take bounds $1 \cdot 10^{-4} \lesssim k \lesssim 1 \cdot 10^{-1}$ (see Section *Estimate of surfactant parameters*), consistently also with values found in the literature for engineered (16) and natural settings (17).

- Boundary layer extending across the microchannel, $\delta \sim O(1)$: Long gratings favor a thick boundary layer since it has more available length to develop. Since $\delta \approx a_3(1 + a_4(Pe/g))^{-1/3}$, $Pe/g \lesssim O(1)$ is needed to have $\delta \sim O(1)$. For microchannel flows, the half-height is in the range $\hat{h} \in [10^{-4}, 10^{-3}]$ m, and the flow velocity $\hat{U} \in [10^{-5}, 10^{-3}]\,\mathrm{m\,s^{-1}}$. Surfactants have diffusivities of order $\hat{D} \sim 1 \cdot 10^{-9}\,\mathrm{m^2\,s^{-1}}$ or smaller (2), yielding $Pe = \hat{h}\hat{U}/\hat{D} \in [1, 10^3]$. Therefore gratings with interface length $g = \hat{g}/\hat{h} \sim 1/\varepsilon \sim 10^2$ the thick boundary layer would be fully developed. For longer gratings, this condition is even more easily satisfied.

- Kinetics are fast compared to diffusion, $Da \gtrsim 1$: For microchannel half-heights and diffusivities as in the points above, and finding the approximate bounds $\hat{\kappa}_a\hat{\Gamma}_m \in [10^{-5}, 10^{-3}]\,\mathrm{m\,s^{-1}}$ in the literature (satisfied for all surfactants except for two in Table 3 of (2)), one has that $Da = \hat{\kappa}_a\hat{\Gamma}_m\hat{h}/\hat{D} \in [1, 10^3]$. Only for $\hat{h}$ considerably smaller than tens of microns one would have very small values of Da.

- Mobilization length larger than the modified depletion length, $L_m \gtrsim L_d^{\mathrm{mod}}$: Since $L_d^{\mathrm{mod}} = \sqrt{Da/(Pe_I Bi)}$, and $L_m = \sqrt{k\,Ma\,Da/Bi}$, to achieve $L_d^{\mathrm{mod}} \lesssim L_m$ one needs $k\,Ma\,Pe_I = k\,n_s\hat{R}\hat{T}\hat{\Gamma}_m\hat{h}(\hat{\mu}\hat{D}_I)^{-1} \gtrsim 1$. We take k in the range described in the points above, $n_s = 2$, $\hat{R} = 8.314\,\mathrm{J/(mol\,K)^{-1}}$, $\hat{T} \sim 300\,\mathrm{K}$, $\hat{\Gamma}_m \sim 10^{-6}\,\mathrm{mol\,m^{-2}}$, $\hat{\mu} = 1 \cdot 10^{-3}\,\mathrm{kg\,(m\,s)^{-1}}$, and $\hat{D}_I \sim 1 \cdot 10^{-9}\,\mathrm{m^2 s^{-1}}$. We then find $k\,Ma\,Pe_I \in [5 \cdot 10^1, 5 \cdot 10^4]$, indicating that $L_d^{\mathrm{mod}} \lesssim L_m$.

- Uniform shear stress, $\gamma(x) \approx \langle\gamma\rangle$: Deviations from this assumption are associated with the 'stagnant cap regime', which requires a dominant interface advection term, with negligible kinetics and diffusion (12). The stagnant cap regime is difficult to achieve for long gratings, since $Bi/\varepsilon \gtrsim 1$ will be easily satisfied, thereby making interface kinetic fluxes significant in the interfacial transport equation (Eq. 2b in the main text). We assume that the channel half-height is in the range $\hat{h} \in [10^{-4}, 10^{-3}]$ m, and that the flow velocity is in the range $\hat{U} \in [10^{-5}, 10^{-3}]\,\mathrm{m\,s^{-1}}$. Based on the review of physicochemical properties of surfactants in Ref. (2) (see their Table 3), we assume $\hat{\kappa}_d$ is in the range $\hat{\kappa}_d \in [10^{-1}, 10^3]\,\mathrm{s^{-1}}$, which covers all but one of the surfactants that they describe. With these bounds, we find $Bi = \hat{\kappa}_d\hat{h}/\hat{U} \in [10^{-2}, 10^5]$. Even with this wide range of parameters, the Biot number remains high enough such that Bi/ε will be at least of order one for gratings that are practical for drag reduction, where $\varepsilon = \hat{h}/\hat{L} \approx \hat{h}/\hat{g} \sim 10^{-2}$ or smaller. The uniform shear stress assumption is also supported by our simulations, which span a wide range of parameters (see Table SII).

SI Dataset S1 (Temprano-Coleto_etal_Simulation_Data.xlsx)
Data for finite-element simulations of the full Eqs. S1a–S1t.

References

1. H Manikantan, TM Squires, Surfactant dynamics: hidden variables controlling fluid flows. *J. Fluid Mech.* **892**, P1 (2020).
2. CH Chang, EI Franses, Adsorption dynamics of surfactants at the air/water interface: a critical review of mathematical models, data, and mechanisms. *Colloids Surf. A* **100**, 1–45 (1995).
3. AJ Prosser, EI Franses, Adsorption and surface tension of ionic surfactants at the air-water interface: review and evaluation of equilibrium models. *Colloids Surf. A* **178**, 1–40 (2001).
4. FJ Peaudecerf, JR Landel, RE Goldstein, P Luzzatto-Fegiz, Traces of surfactants can severely limit the drag reduction of superhydrophobic surfaces. *Proc. Nat. Acad. Sci. USA* **114**, 7254–7259 (2017).
5. G Bolognesi, C Cottin-Bizonne, C Pirat, Evidence of slippage breakdown for a superhydrophobic microchannel. *Phys. Fluids* **26**, 082004 (2014).
6. D Schäffel, K Koynov, D Vollmer, HJ Butt, C Schönecker, Local flow field and slip length of superhydrophobic surfaces. *Phys. Rev. Lett.* **116**, 134501 (2016).
7. D Song, et al., Effect of a surface tension gradient on the slip flow along a superhydrophobic air-water interface. *Phys. Rev. Fluids* **3**, 033303 (2018).
8. JR Philip, Flows satisfying mixed no-slip and no-shear conditions. *Z. Angew. Math. Phys.* **23**, 353–372 (1972).
9. CJ Teo, BC Khoo, Analysis of stokes' flow in microchannels with superhydrophobic surfaces containing a periodic array of micro-grooves. *Microfluid. Nanofluidics* **7**, 353 (2009).
10. E Lauga, HA Stone, Effective slip in pressure-driven Stokes flow. *J. Fluid Mech.* **489**, 55–77 (2003).
11. JR Landel, et al., A theory for the slip and drag of superhydrophobic surfaces with surfactant. *J. Fluid Mech.* **883**, A18 (2020).
12. LG Leal, *Advanced Transport Phenomena: Fluid Mechanics and Convective Transport Processes*, Cambridge Series in Chemical Engineering. (Cambridge University Press), (2007).
13. I Miranda, et al., Properties and applications of PDMS for biomedical engineering: A review. *J. Funct. Biomater.* **13**, 2 (2021).

14. H Li, et al., Three-dimensional backflow at liquid–gas interface induced by surfactant. *J. Fluid Mech.* **899**, A8 (2020).
15. WM Haynes, *CRC handbook of chemistry and physics.* (CRC press), (2014).
16. SSD Carter, et al., PDMS leaching and its implications for on-chip studies focusing on bone regeneration applications. *Organs-on-a-Chip* **2**, 100004 (2020).
17. AA Frossard, et al., Properties of seawater surfactants associated with primary marine aerosol particles produced by bursting bubbles at a model air–sea interface. *Environ. Sci. & Technol.* **53**, 9407–9417 (2019).
